# Structural Basis for the Bidirectional Activity of *Bacillus* nanoRNase NrnA

**DOI:** 10.1038/s41598-017-09403-x

**Published:** 2017-09-11

**Authors:** Brad J. Schmier, Claudiu M. Nelersa, Arun Malhotra

**Affiliations:** 1 Department of Biochemistry and Molecular Biology, University of Miami Miller School of Medicine, PO Box 016129, Miami, FL 33101-6129 USA; 2 Present Address: Molecular Biology Program, Sloan-Kettering Institute, New York, NY 10065 USA

## Abstract

NanoRNAs are RNA fragments 2 to 5 nucleotides in length that are generated as byproducts of RNA degradation and abortive transcription initiation. Cells have specialized enzymes to degrade nanoRNAs, such as the DHH phosphoesterase family member NanoRNase A (NrnA). This enzyme was originally identified as a 3′ → 5′ exonuclease, but we show here that NrnA is bidirectional, degrading 2–5 nucleotide long RNA oligomers from the 3′ end, and longer RNA substrates from the 5′ end. The crystal structure of *Bacillus subtilis* NrnA reveals a dynamic bi-lobal architecture, with the catalytic N-terminal DHH domain linked to the substrate binding C-terminal DHHA1 domain via an extended linker. Whereas this arrangement is similar to the structure of RecJ, a 5′ → 3′ DHH family DNase and other DHH family nanoRNases, *Bacillus* NrnA has gained an extended substrate-binding patch that we posit is responsible for its 3′ → 5′ activity.

## Introduction

The synthesis and degradation of RNA are fundamental processes regulating gene expression. Many processive exoribonucleases leave nanoRNA (RNA fragments 5 nucleotides or smaller) limit products as they approach the ends of their substrate RNA. NanoRNAs are also generated in other biological processes including abortive transcription initiation^1–3^ and the cleavage of cyclic dinucleotide second messengers^4–7^. It has been demonstrated that nanoRNAs can prime transcription initiation and regulate gene expression^2, 8–10^. Moreover, accumulation of the pGpG nanoRNA in *P*. *aeruginosa* stimulates c-di-GMP signal transduction and consequent overproduction of biofilms and extracellular polymers^5, 6^.

NanoRNAs are degraded to mononucleotides by specialized RNases^11^. In *E. coli*, oligoribonuclease (Orn) degrades nanoRNA to mononucleotides^12, 13^, and is the only essential exoribonuclease in that organism^11^. Orn homologs have been identified in some bacteria and all eukarya examined to date^14, 15^. However, many bacteria (*e.g*. Firmicutes, Bacteroidetes) and archaea lack *orn* homologs^16^. In the Firmicute *B. subtilis*, the *ytqI* gene product was shown to be a major nanoRNA turnover enzyme, and was renamed NanoRNase A (NrnA)^16–18^. Homologs of *nrnA* are widespread in organisms lacking *orn* ^16^, which suggests that *nrnA* represents the founding member of a new class of nanoRNases.

NrnA belongs to the DHH family of phosphoesterases^19^ that includes *Drosophila* prune protein, *S. cerevisiae* exopolyphosphatase, *E. coli* RecJ exonuclease, *Bacillus* pyrophosphatase, and *Mycobacterium* cyclic di-NMP phosphodiesterase. Proteins in this family have a conserved N-terminal catalytic domain with four distinct sequence motifs (I–IV) and an associated C-terminal domain that varies between family members (Supplementary Fig. S1). Each motif has at least one conserved aspartate residue, with Motif III harboring the DHH sequence after which the family is named. The DHH motif and several of the conserved acidic residues coordinate two divalent cations that activate a water molecule for nucleophilic attack on the phosphodiester backbone^19^. NrnA has a C-terminal DHHA1 (DHH-associated domain 1) that also is present in alanyl-tRNA synthetase (AlaRS) and RecJ, and is predicted to play a role in nucleic acid binding^20–22^.

NrnA was originally described as a (i) 3′ → 5′ exoribonuclease with a preference for nanoRNA substrates; and (ii) a 3′ pAp phosphatase^16^. *Bacillus nrnA* complements both an *E. coli orn* ^ts^ mutant and a deletion of *cysQ*, a gene responsible for pAp turnover^16^. However, NrnA is homologous to RecJ, a 5′ → 3′ DHH family DNase, and it has been suggested that NrnA is a 5′ → 3′ exonuclease based on mass spectrum analysis of *in vitro* generated reaction products^23, 24^. Structures of nanoRNases from *Bacteroides fragilis* ^24^, *Mycobacterium smegmatis* ^25^, and *Mycobacterium tuberculosis* ^7^ have yielded important insights into the 2-metal ion mechanism of the DHH domain and the nucleotide binding pocket of the DHHA1 domain, but have not cleared up confusion regarding the polarity of NrnA.

We show here that *Bacillus* NrnA is a bidirectional nuclease that can degrade RNA fragments from either the 3′ or the 5′ end. We have determined crystal structures of *B. subtilis* NrnA in its apo form, as well as complexed with substrates. These structures reveal a dynamic bi-lobed molecule with the DHH catalytic domain and the DHHA1 substrate binding domains connected through an extended linker. Whereas this overall arrangement is very similar to the structure of RecJ, *Bacillus* NrnA also has a positively charged patch on its C-terminal domain that we posit forms a nanoRNA binding site responsible for its 3′ → 5′ activity. Comparison of the NrnA and RecJ structures allows us to propose a mechanism of how these two DHH family phosphoesterases can function as both 3′ → 5′ and 5′ → 3′ exonucleases.

## Results

### Sequence and domain analysis of NrnA

**Figure 1:**
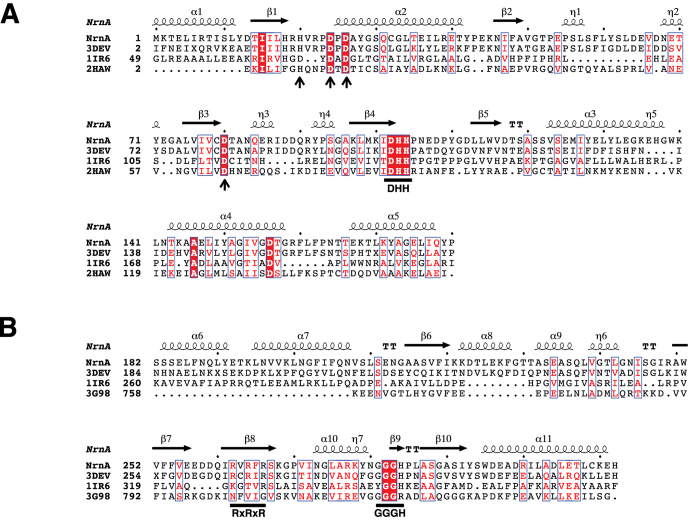
Sequence motifs of NrnA. (**A**) Structure based sequence alignment of the N–terminus DHH domain of NrnA and selected structural homologs (Sh1221 protein from *Staphylococcus haemolyticus* (PDB ID: 3DEV), RecJ exonuclease from *Thermus thermophilus* (PDB ID: 1IR6), and family II inorganic PPase from *Bacillus subtilis* (PDB ID: 2HAW)). Active site resides are marked by arrows and the DHH motif. Identical residues are highlighted by a red background, while regions of similarity are boxed. (**B**) Structure based alignment of the C-terminal DHHA1 domain of NrnA and selected structural homologs (3DEV, 1IR6, and the C-Ala domain of Alanyl-tRNA synthetase from *Aquifex aeolicus* (PDB ID: 3G98)). The RxRxR and GGGH motifs that play a role in substrate binding are marked.

NrnA is a member of the DHH family of phosphoesterases that includes enzymes with a broad range of substrate specificities^19^. Structure based multiple sequence alignment of several putative bacterial nanoRNases shows that the characteristic motifs I-IV of the DHH family^19^ are present in NrnA homologs (Fig. 1A and Supplementary Fig. S1). The C-terminal DHHA1 domain of NrnA homologs contains a highly conserved GGGH-x-x-ASG motif (Supplementary Fig. S1). Similar GG motifs are present in other proteins with DHHA1 domains such as AlaRS and RecJ (Fig. 1B), but it has been suggested that exonucleases in this family (RecJ and NrnA) may extend this motif to terminate with a histidine (H284 in *B. subtilis* NrnA) for substrate recognition^7, 22, 24–26^. *Bacillus type* NrnA homologs also contain an R-x-R-x-R motif (R262, 264, and 266 in *B. subtilis* NrnA) that is only partially conserved in other DHHA1 domains (only the arginine equivalent to R266 is conserved in RecJ; Fig. 1B and Supplementary Fig. S1).

### Structure of NrnA

*Bacillus* NrnA was crystallized in the *P*2_1_ space group with four molecules per asymmetric unit^27^. The 2.0 Å structure of native apo-NrnA was solved using phases obtained by molecular replacement with the structure of a homologous unpublished *S. haemolyticus* protein (PDB ID: 3DEV). Structures of an NrnA active site mutant (His103 mutated to Ala–H103A) were also solved bound with several ligands (pAp with and without metal ions, and pAA*A, where * represents a poorly hydrolysable phosphorothioate linkage), to better understand the mechanism of this nuclease. These structures were solved by molecular replacement using only the DHH domain from the native apo NrnA structure as a search model. Data collection and refinement statistics for all structures are presented in Supplementary Table S1.

**Figure 2:**
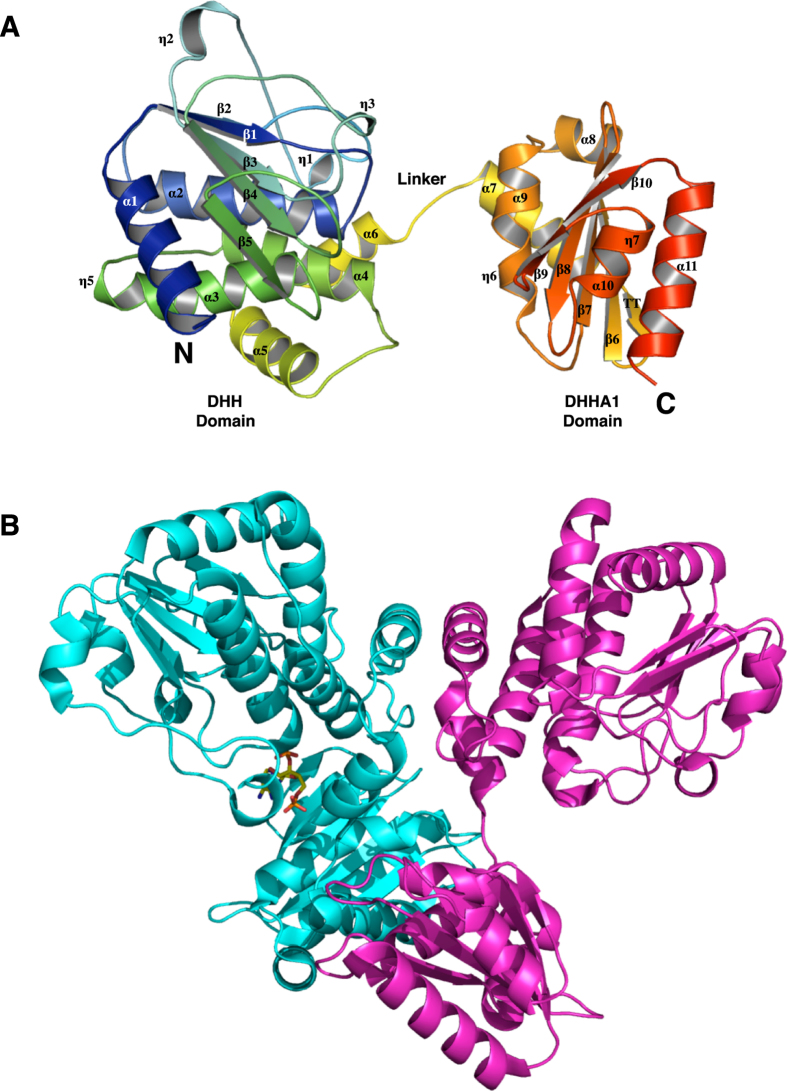
Structure of *B. subtilis* NrnA. (**A**) The overall structure of *Bacillus* NrnA in a ribbon representation (PDB ID: 5J21). Image was generated using Pymol^47^. (**B**) Structure of the *B. subtilis* NrnA homodimer from NrnA-H103A:pAp (PDB ID: 5IUF). The two monomers are colored cyan and pink, and the bound ligand is shown as sticks.

The *B. subtilis* NrnA structure comprises two globular domains connected by a linker region (residues 178–194) that consists of a short α-helix bordered by two loops (Fig. 2A). The N-terminal DHH catalytic domain adopts a mixed α/β fold featuring a 5-stranded parallel β-sheet at its core. Residues from the DHH motifs come together to form a surface-exposed active site that faces the C-terminal substrate-binding domain. The C-terminal DHHA1 domain of NrnA also adopts an α/β fold with a central mixed β-sheet and helices on both the apical and basal sides (Fig. 2A). The R-x-R-x-R motif lies on strand β8 of the central β-sheet, while the GGGH motif lies at the interface of a loop and the terminal β-strand; both these motifs face the DHH domain and the active site.

NrnA from *B. fragilis, M. smegmatis* and *M. tuberculosis* are reported to be dimeric, and *Bacillis* NrnA in our crystals packs in a similar manner (Fig. 2B). The dimer interface in *Bacillus* NrnA covers about 1600 Å^2^, 11% of the total surface area of the molecule, and is centered around helices α5 and α9. Whether dimerization is a consequence of crystal packing or is the physiologically active entity is unclear. In either case, the NrnA active site directly faces the DHHA1 domain of the same monomer and away from the dimerization interface, indicating the catalytic cycle of an individual nanoRNA occurs within one monomer.

The *B. subtilis* NrnA structure is similar to other DHH family proteins. Top structural homology matches to NrnA, identified using the DALI server, include *S. haemolyticus* Sh1221 protein (Z-score of 38.3; PDB ID: 3DEV; sequence identity: 48%), *M. tuberculosis* Rv2837c c-di-NMP phosphodiesterase (Z-score of 27.1; PDB ID: 5CEU; sequence identity: 22%), *B. subtilis* inorganic PPase (Z-score of 18.8; PDB ID: 1K23; sequence identity: 16%), and the *Thermus thermophilus* RecJ exonuclease (Z-score of 17.8; PDB ID: 1IR6; sequence identity: 14%).

### The C-terminal DHHA1 domain is mobile

**Figure 3:**
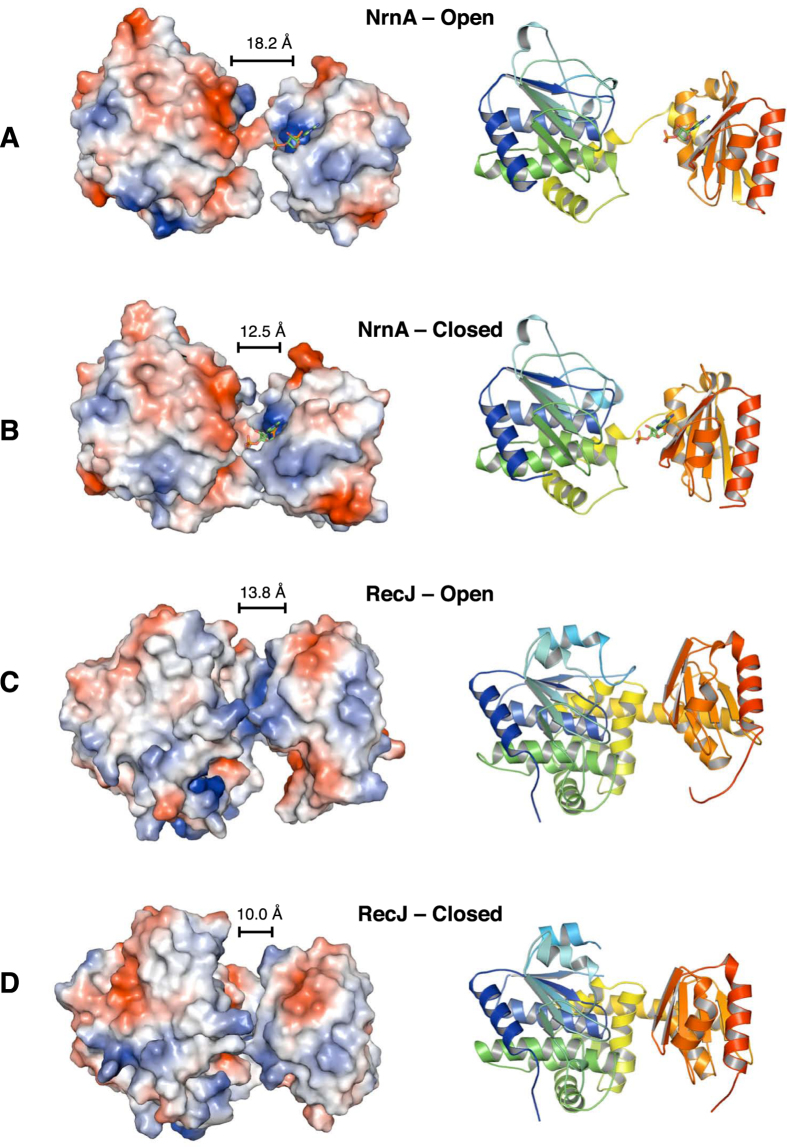
Mobility of the NrnA DHHA1 substrate binding domain. (**A**,**B**) Two arrangements of the NrnA domains are seen in our structures: open (A) and closed (B). The panels show two different monomers in the asymmetric unit of NrnA-H103A:pAp crystal structure. (**C**,**D**) Similar mobility of the DHHA1 domain is also seen in structures of the DNA exonuclease, RecJ. Panel C shows an open form (PDB ID: 1IR6), while the closed form is seen in PDB ID: 2ZXP (panel D). For clarity, only DHH and DHHA1 domains are shown for RecJ. Panels show the same orientation of the DHH domain with only the DHH catalytic motif residues superposed. Structures are shown as a ribbon diagram (right side), and as a molecular accessible surface colored by electrostatic potential using Pymol (left side).

The four NrnA molecules in the *P*2_1_ asymmetric unit of our crystal structures display variation in the orientation of the C-terminal DHHA1 domain with respect to the DHH domain, and the distances between Cα of H284 (at the end of the GGGH motif) and Cα of H104 (in the DHH active site) can vary from 11.9 to 19.3 Å. These variations can be classified broadly as an “open” state (Figs 2 A and 3A), in which the C-terminal domain has rotated away from the DHH domain, exposing the catalytic center, and as a “closed” state, in which the two domains are in closer proximity and the catalytic center is less accessible (Fig. 3B). Similar conformational variations are seen in the structures of the DHH family enzymes RecJ and PPase, and also in homologous nanoRNases, where it has been suggested that the C-terminal domain is dynamic and may play a role in bringing substrate to the N-terminal catalytic domain within an individual monomer^7, 22, 24, 25, 28^.

In structures of NrnA with the bound ligand, pAp, the C-terminal domain rotates more than 6 Å towards the catalytic domain between the open and closed states (Fig. 3A and B). At the center of the mobile region is a positively-charged RNA-binding pocket, supporting the notion that mobility of the DHH associated domain aids in substrate transport to the catalytic domain. Interestingly, ligand bound structures of NrnA crystals soaked with a 3 mer pAA*A are observed only in the closed conformation (spacing of 12.2 Å between Cα of H284 and Cα of H104), suggesting that nanoRNA substrates may facilitate interactions between the two domains (Supplementary Fig. S5). A similar case is observed in two apo-RecJ structures (Fig. 3C and D), where the GGH motif rotates by 7.6 Å towards the catalytic domain^22^, and also other DHH family nanoRNases (Supplementary Fig. S2)^7, 24, 25^.

### NrnA active site

**Figure 4:**
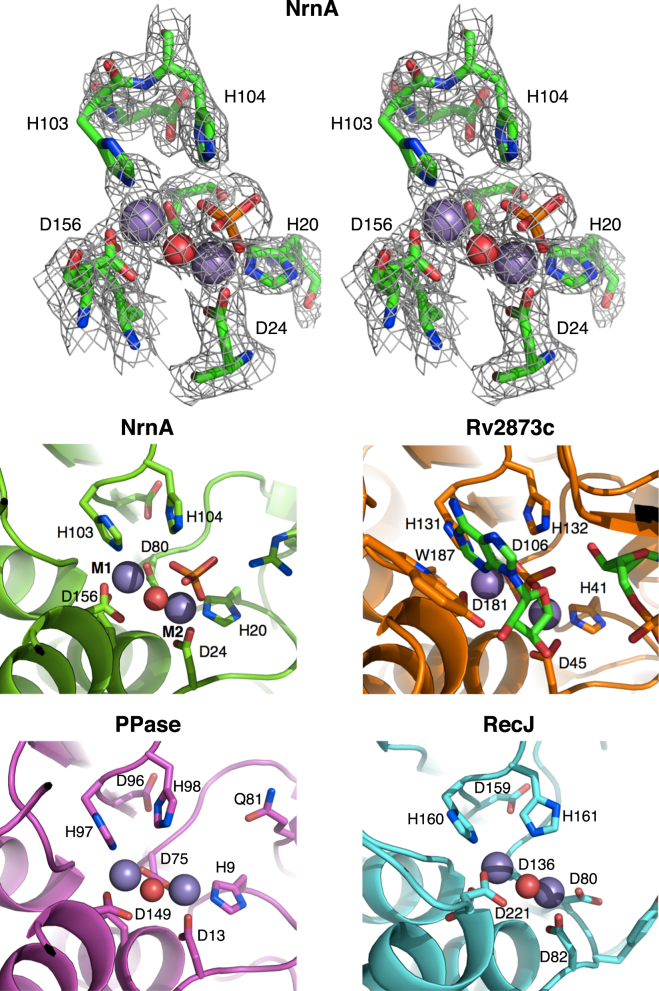
Details of the NrnA active site. Top left panel shows 2*Fo*-*Fc* electron density in stereo around the *Bacillus* NrnA active site from the H103A:Mn:pAp structure with two bound Mn^2+^ ions (purple spheres) shown at full Van der Waals radii. M1 (*left*) and M2 (*right*) are separated by 3.8 Å and coordinate several Asp and His residues and a central water molecule (red) shown at 0.75x Van der Waals radii. A product phosphate group observed in the H103A:Mn:pAp structure is also shown. 2*Fo*-*Fc* electron density is contoured at 5 *σ* around M1 and M2, and 1.5 *σ* around all other atoms. The native H103 is modeled in lieu of Ala in the H103A mutant. The middle left panel shows a cartoon representation of the same H103A:Mn:pAp active site from *Bacillus* NrnA. The middle right panel shows the active site of Rv2873c from *M. tuberculosis* after cleavage of pApA (PDB ID: 5JJU). The bottom two panels show the active sites of the family II inorganic PPase from *Bacillus subtilis* (PDB ID: 1K23) and *T. thermophilus* RecJ (PDB ID: 2ZXP). All representations are shown in the same orientation.

The NrnA active site is formed by four aspartate residues (D24, D26, D80 and D156) and three histidines (H103, H104 and H20) that combine to generate a shallow cleft in the DHH domain. To investigate metal binding at the NrnA active site, NrnA H103A (purified in the presence of 2 mM EDTA) was crystallized as described and soaked with 10 mM MnCl_2_ and 10 mM pAp^27^. Strong 2*Fo*-*Fc* electron density (ranging from 9.0 *σ* to 6.5 *σ*) was seen at two positions in each active site for all four molecules in the asymmetric unit. This density was modeled as two Mn^2+^ ions present at full occupancy (Fig. 4). Two bound Mn^2+^ ions at the NrnA active site are characteristic of DHH family active sites including those of inorganic PPase and RecJ, as well as NrnA homologs^7, 21, 22, 24, 26, 28^. Coordination of metal 1 (M1) occurs with H103 of the DHH motif and the aspartate carboxylates of D26, D80 (bidentate ligand), and D156. Metal 2 (M2) also coordinates with aspartate carboxylates including of D24, as well as a histidine, H20, which is substituted by an Asp in the RecJ active site (Fig. 4)^19, 21, 22, 26^. A difference map contoured at 5.0 *σ* revealed the presence of an ordered water molecule between M1 and M2 that provides additional oxygen ligands to the metal ions and may be activated for nucleophilic attack of the incoming substrate. H104, the second histidine in the signature DHH motif, does not coordinate with M2 and is therefore accessible for interaction with the substrate’s phosphodiester backbone.

A distinctive feature of NrnA is its additional 3′ pAp phosphatase activity^16^. In the crystals of NrnA H103A soaked with both MnCl_2_ and pAp, electron density corresponding to the full pAp ligand is not seen, presumably because the mutant still retains some residual activity. Instead, additional tetrahedral electron density outside M2 is clearly visible in all four chains when 2*Fo*-*Fc* and *Fo*-*Fc* electron density maps are contoured at 1.75 σ and 3.50 σ respectively. This density, modeled as phosphate (Fig. 4), may correspond to the 3′ phosphate excised from pAp after pA has diffused away from the exposed DHH active site, and is located in the same position as the scissile phosphate of pApA bound to *M. tuberculosis* Rv2837c (Fig. 4) and the *D. radiodurans* RecJ bound to ssDNA^7, 26^.

### pAp bound complex reveals the NrnA nanoRNA binding site

**Figure 5:**
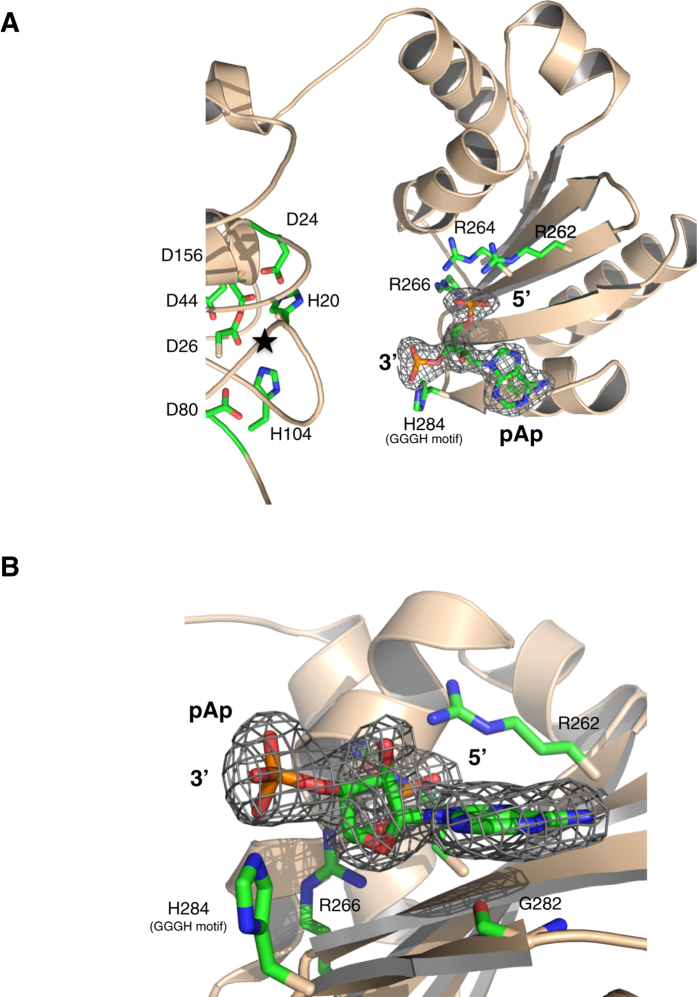
Substrate binding site of NrnA (**A**) A view of pAp bound on NrnA in the H103A:pAp complex crystallized in absence of metal ions. The NrnA active site on the DHH domain is highlighted by a star. (**B**) Close-up view of pAp bound on the NrnA DHHA1 domain from the H103A:pAp complex crystallized in absence of metal ions. The mesh represents 2*Fo*-*Fc* electron density contoured at 1.0 *σ* around the bound ligand. Figures were created in Pymol^47^.

To better understand pAp binding, we soaked pAp into NrnA-H103A crystals in the presence of EDTA and with no metal added. The structure of the H103A:pAp complex (Supplementary Table S1) shows clear density for the non-hydrolyzed substrate bound at the C-terminal DHHA1 domain with its 3′ phosphate facing the active site (Fig. 5A and B) in two of the four NrnA monomers in the asymmetric unit (Supplementary Table S1). The substrate lies in a shallow, positively charged groove containing several basic residues (Fig. 3A and B). Three arginines, observed in close homologs of *Bacillius* NrnA (R262, R264, and R266) line this groove, and form ion-pair interactions between the side chain guanido groups and the nonesterified oxygen atoms of the substrate’s 5′ phosphate (Fig. 5A and B). R262 and R264, which lie furthest from the active site, bind distinct oxygens of the 5′ phosphate. In the pAp-bound structure, the R262 side chain has rotated more than 180° (around the CD—NE bond) from its apo-structure orientation to form ionic interactions with the pAp 5′ phosphate. R266 has also rotated away from the solvent channel, compressing in structure to form contacts with the 5′ phosphate. Finally, a H-bond network mediated by a bound water molecule is formed between the backbone carbonyl of V263, the NE and guanidino group of R262, and the 5′ phosphate group of the substrate (Supplementary Fig. S3).

The conformation of the bound pAp substrate reveals the adenosine in the anti conformation and the ribose with a C2′ endo-pucker (Fig. 5). In both open and closed states of the pAp bound NrnA, the adenosine base is stabilized by backbone interactions with the G282 carbonyl oxygen, a part of the highly conserved GGGH motif. These results indicate that both the R-x-R-x-R motif, and the GGGH motifs in the *Bacillus* NrnA DHHA1 domain form a substrate binding site optimized for nanoRNAs.

To examine binding of larger nanoRNA substrates, crystallization of NrnA with several di- and tri-nucleotide ligands were carried out. In most of these trials, the substrate was likely hydrolyzed due to residual activity of the H103A mutant as no additional density was visible (Supplementary Fig. S4). However, the crystal structure of NrnA H103A:pAA*A complex (pAA*A, where * represents a phosphorothioate linkage, soaked into H103A in presence of EDTA and in the absence of metal; Supplementary Table S1) did reveal additional density. Electron density corresponding to a pA mononucleotide was seen in the same location as pAp in the H103A:pAp structure (Supplementary Fig. S5A), but it was not continuous enough to build the full ligand. However, we did see a clear region of additional density between H284 and a solvent exposed phenylalanine from the catalytic domain (F162). Polder omit maps^29^ centered on F162 and contoured at 2.5 σ indicate the density represents an adenosine from the pAA*A ligand (Supplementary Fig. S5B). As in the case of the H103A:pAp structure, the additional pAA*A density is seen only in two of the four monomers in the asymmetric unit. Unlike the H103A:pAp structure, the H103A:pAA*A structure is in a closed conformation, suggesting that a longer substrate may stabilize interactions between the two NrnA domains. F162 is structurally equivalent to W187 in Rv2873c, a residue observed to stack with the AMP adenosine at the 3′-end of a cleaved dinucleotide in a closed conformation of an Rv2873c monomer (Supplementary Fig. S5C).

### NrnA is a bi-directional exoribonuclease

The NrnA-H103A:pAp structure shows the pAp ligand bound at the DHHA1 nanoRNA binding site, but several Å away from the catalytic site (Fig. 5). However, the 3′ end of the ligand is closest to the DHH active site, and it is easy to envision how nanoRNA is presented for hydrolysis at its 3′ end (see discussion). This is in agreement with the reported 3′ → 5′ activity of NrnA on nanoRNAs^16, 18, 25^. However, a few studies have reported that NrnA has 5′ → 3′ activity on an A11 substrate^23, 24^. Another DHH family nuclease closely homologous to NrnA in both sequence and structure (Figs 1 and 2), RecJ, is also a well characterized 5′ → 3′ DNase.

**Figure 6:**
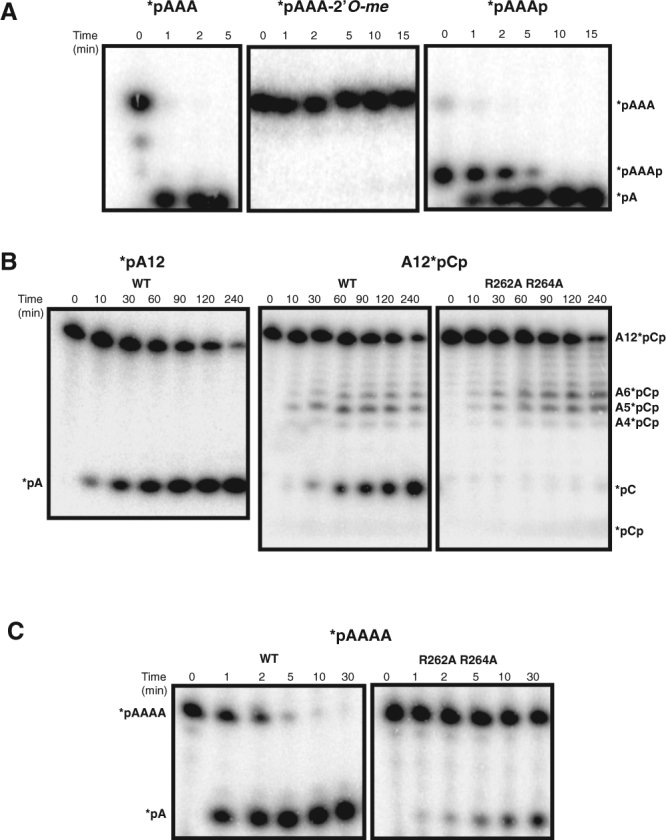
NrnA is a bi-directional exoribonuclease. (**A**) NrnA activity on 3-mer RNA oligonucleotides. WT NrnA activity is compared on *pA3, *pA3-2′*O-*me with only the 3′ nucleotide methylated, and *pA3p, radiolabeled at their 5′ ends with ^32^P (*) using T4 polynucleotide kinase and [γ^32^P]-ATP. Equivalent amounts of NrnA (0.285 μM) and substrate (1 μM) were used in all reactions. (**B**) NrnA activity on 12-mer RNA oligonucleotides. (*left*) WT NrnA (2.85 μM) was assayed on A12 (1 μM) labeled at its 5′ end. WT (*center*) and R262A R264A (*right*) NrnA (2.85 μM) were assayed on A12 (1 μM) labeled at its 3′ end with *pCp by T4 RNA ligase. (**C**) WT (0.1 μM) and R262A R264A (0.1 μM) NrnA were compared on *A4 (5 μM). Black dividing lines demarcate distinct gels or sections of a gel.

To resolve the directionality of NrnA activity, we carried out a series of biochemical assays. We first examined whether NrnA could degrade RNA in a 3′ → 5′ manner using as substrate a three nt oligomer (A3) labeled at its 5′ end with ^32^P (Fig. 6A). NrnA rapidly degrades this molecule generating only ^32^P-AMP with no intermediates. To confirm the 3′ → 5′ exonuclease activity, we examined whether the enzyme was sensitive to modifications at the substrate’s 3′ end. A3 modified with a 2′-O-methylation at its 3′-terminal nucleotide (*pAAA-2′*O*-me) was tested in an identical reaction to that described above, and as can be seen, the modification completely inhibits NrnA activity (Fig. 6A). NrnA is also sensitive to the presence of a 3′ phosphate, which reduces its activity relative to that of a 3′ hydroxyl (Fig. 6A). Furthermore, when challenged with a trinucleotide blocked at the 5′ end with a fluorescent dye (Dy647-A3), NrnA exonucleolytically degrades the substrate in a distributive manner, removing individual mononucleotides from the 3′ end (Supplementary Fig. S4A). These results clearly demonstrate that NrnA has 3′ → 5′ activity on nanoRNA.

NrnA is homologous to the DNase, RecJ, sharing a similar linear domain architecture between its catalytic (DHH) and substrate binding (DHHA1) domains (Supplementary Fig. S1). However, RecJ is a 5′ → 3′ exonuclease which requires single-stranded DNA 6–7 nt or longer for effective binding^26, 30, 31^. In view of its homology to RecJ, we examined whether NrnA also might display 5′ → 3′ activity on longer substrates similar to those preferred by RecJ. Accordingly, NrnA was assayed using A12 radiolabeled at either its 5′ end with ^32^P (*A12) or its 3′ end with ^32^pCp (A12*pCp). While higher enzyme levels and longer incubation periods were needed (Fig. 6B), we observe clear evidence of degradation from the 5′ end. NrnA removes the 5′ label from *A12, and its action on the 3′ labeled substrate generates intermediates that could only be produced if degradation initially proceeds in the 5′ → 3′ direction. The presence of intermediates suggests that in the 5′ → 3′ direction, the enzyme acts in a distributive fashion. Since no fragments shorter than 5 nt are seen, but *pC is produced, we propose that the short fragments dissociate and are degraded via the 3′ → 5′ activity. The phosphatase activity is responsible for converting the *pCp produced to *pC. The rate of *pA appearance from *pA12 by WT NrnA is roughly 3-fold faster than the appearance of *pC from A12*pCp, consistent with a 5′ → 3′ mechanism of decay that first removes the 5′ mononucleotide (Fig. 6B).

As suggested by our structural data, R262 and R264 are likely to play an important role in binding nanoRNA substrates. These residues are conserved among many NrnA homologs, but are absent in RecJ and nanoRNases that prefer dinucleotides (Fig. 1B)^7, 18, 22, 24–26^. Indeed, when these residues are mutated to alanine, the mutant NrnA (R262A R264A) is 9-fold less active than WT enzyme on a *pA4 substrate (Fig. 6C and Supplementary Fig. S4B) but maintains 5′ → 3′ activity on A12*pCp, accumulating the same intermediates as WT NrnA (Fig. 6B). However, no *pC is generated, consistent with loss of the 3′ → 5′ activity. We propose that R262 and R264 play an important role in conferring *Bacillus* NrnA with a 3′ → 5′ activity, not seen in the homologous RecJ. An early report on RecJ mentions a weak 3′ → 5′ DNase activity that has not been further characterized^30^.

## Discussion

Based on sequence and domain organization, NrnA is a typical member of the DHH family of phosphoesterases (Supplementary Fig. S1). This sequence homology is also reflected in the structure of *Bacillus* NrnA, which is similar to other DHH family enzymes, including the DNA exonuclease, RecJ (Fig. 3). However, this similarity is not reflected in the activity of these two enzymes: RecJ is a well characterized 5′ → 3′ exonuclease active on single-strand DNA substrates longer than 6 nucleotides, while NrnA has 3′ → 5′ activity on very short RNA substrates, and weaker 5′ → 3′ activity on longer RNA substrates (Fig. 6)^16, 30^.

**Figure 7:**
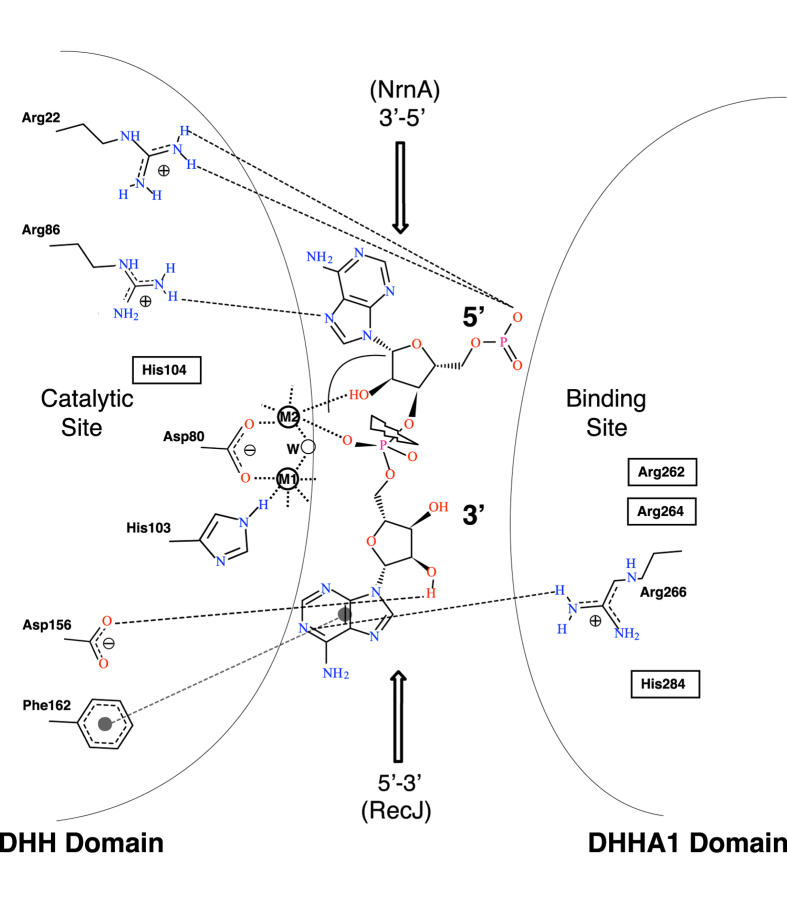
A schematic representation of RNA hydrolysis by NrnA from either the 3′ or the 5′ end. If the 3′ nucleotide of a RNA chain is placed at the active site and the chain extends at the top, hydrolysis will proceed in the 3′ → 5′ direction (arrow pointed down). On the other hand, if a RNA chain is placed so that the 5′ nucleotide is positioned at the active site, hydrolysis will proceed in the 5′ → 3′ direction (arrow pointed up; this is the primary activity seen in RecJ). The figure shows a dinucleotide (pApA) in the catalytic site of NrnA poised for hydrolysis (jagged arrow), with interacting residues from either the catalytic DHH domain (left side of the panel) or the binding DHHA1 domain (on the right). H-bond interactions (based on a dinucleotide modeled into the H103A:Mn:pAp co-crystal structure; see Supplementary Fig. S6) are shown as dashed lines, while the stacking interaction of the 3′ base with F162 is shown with a dashed line bound by shaded circles. The two metal ions at the active site are represented as circles marked M1 and M2, and the activated water is marked w. Parts of this figure were created using PoseView^48^.

Can the same structural architecture give rise to nucleases that work with opposite polarities or have bi-directional activity, as appears to be the case for NrnA? An answer to this apparent conundrum comes from the fact that when a nucleic acid chain is being hydrolyzed exonucleolytically into mononucleotides, its phosphodiester backbone has to be positioned for hydrolysis in the same manner for cleavage from either the 5′ or the 3′ direction (see schematic in Fig. 7, with a dinucleotide positioned for hydrolysis at the two-metal active site). If a nucleic acid substrate is positioned so that the 5′ end is placed at the cleavage site, exonuclease activity will proceed in the 5′ → 3′ direction; on the other hand, if the enzyme presents a nucleic acid chain in a manner that the 3′ end is positioned at the cleavage site, the exonuclease activity will be 3′ → 5′. In both cases, the bond being broken is between the 3′ oxygen and the phosphate group, resulting in a mononucleotide product and a nucleic acid chain shortened at either the 3′ OH end or the 5′ monophosphate end. This observation is consistent with structures of Rv2837c post-cleavage of the linear pApA dinucleotide^7^ (Fig. 4 and Supplementary Fig. S5C) and *Deinococcus* RecJ bound to ssDNA^26^.

Based on our structural and biochemical data, we propose that NrnA binds nanoRNA substrates in a manner that efficiently cleaves them in a 3′ → 5′ manner, while still retaining some of the binding characteristics of RecJ and thus exhibiting residual 5′ → 3′ nuclease activity.

The different co-crystal structures (Supplementary Table S1) allow us to piece together an overall model of *Bacillus* NrnA’s substrate binding and catalysis of nanoRNA substrates (Fig. 7). Our model describes the catalytic cycle of an NrnA monomer, which our structures and those of other DHH family nanoRNases indicate as the logical enzymatic unit^7, 24, 25^ In the apo-state, NrnA likely fluctuates between the open and closed conformations (Fig. 3). Binding of its preferred substrate, a 3–4 nt RNA molecule, occurs on a positively charged patch of the C-terminal domain composed of the R-x-R-x-R motif specific to *Bacillus* type DHH family nanoRNases, as well as the GGH motif, also seen in other DHHA1 domains including RecJ. The R-x-R-x-R motif (R262, R264, R266) is responsible for binding the phosphodiester backbone, while backbone carbonyls of the GGGH motif provide base stabilization (Fig. 5). The terminal histidine of the GGGH motif (H284) forms contacts with the phosphodiester backbone towards the 3′ end of the substrate (Fig. 5). We can model a pApA dinucleotide at the binding site of a closed NrnA by extending the coordinates of the observed pAp in the 3′ direction (Supplementary Fig. S6). In this model, the 3′ terminal base can participate in a base stacking interaction with F162, a conserved solvent-exposed aromatic residue from the catalytic domain, as suggested by electron density observed in the H103A:pAA*A co-crystal structure (Supplementary Fig. S5B).

Once the substrate is bound to the DHHA1 domain, the closed conformation of NrnA is stabilized. The NrnA closed conformation positions the bound nanoRNA substrate very close to the DHH active site of the same monomer, allowing the substrate to be transported from the DHHA1 domain to the DHH domain. Positioning of the nanoRNA substrate in a conformation conducive for hydrolysis is likely aided by positive patches above the DHH active site (see below).

We can take the product phosphate seen at the active site in our H103A:Mn:pAp structure, the 3′ nucleotide from the H103A:pAp structure, and the planar density outside F162 observed in the H103A:pAA*A to build a composite model of the pApA dinucleotide in the *B. subtilis* NrnA active site (Fig. 7 and Supplementary Fig. S6). In this model, the 5′ end of the dinucleotide is stabilized by interactions with R86 and R22 on the DHH domain (Fig. 7). The adenosine of the 3′ terminal nucleotide forms strong base stacking interactions with F162, orienting the 3′ terminal nucleotide for catalysis (Supplementary Fig. S5), consistent with observations of structurally equivalent W187 of the Rv2873c dinucleotide bound structures (Fig. 4 and Supplementary Fig. S5C)^7^. The 2′ and 3′ hydroxyl of the 3′ terminal ribose H-bonds with DHH domain residues G155, D156, and N164. Disruption of these contacts may explain NrnA’s sensitivity to 3′ terminal ribose modifications on nanoRNA (Fig. 6A). In summary, NrnA functions as a 3′ → 5′ nanoRNase due to an expanded, C-terminal RNA binding surface that accommodates nanoRNA, properly positioning the 3′ terminal nucleotide for exonucleolytic cleavage.

While the RecJ DHHA1 domain maintains a GGH motif and the equivalent of R266, it lacks the expanded nanoRNA-binding pocket seen in *Bacillus* NrnA. However, RecJ does have many conserved positively charged residues on the opposite end of the active site (below the equivalent of F162 and H284 in NrnA). These residues, which come from both the DHH and DHHA1 domains, define a substrate binding path that positions a long DNA substrate with its 5′ end at the DHH active site^21, 22, 26^.

Comparing our structures of NrnA with RecJ, it is clear that these two enzymes hold nucleic acid substrates in opposite orientations (see arrows in Fig. 7 and Supplementary Fig. S6). NrnA has a short binding domain above the DHH active site, and thus presents the 3′ end of nanoRNA for exonucleolytic cleavage. RecJ, on the other hand, has an extended substrate binding channel below the DHH active site, and thus presents single-stranded DNA substrates for 5′ → 3′ cleavage (Supplementary Fig. S6D). RecJ harbors an additional C-terminal OB domain that helps bind longer substrates and also 5′-ssDNA-dsDNA junctions. Structures of the *D. radiodurans* RecJ with DNA bound suggest that binding by the OB domain helps thread the 5′-ssDNA overhangs to the DHH active site along the 5′ → 3′ path and may oblige the protein to persist as a monomer^25, 26^ (Supplementary Fig. S6D). The region occupied by the RecJ OB domain is reserved in NrnA for the second subunit of the homodimer.

Based on our biochemical data (lack of intermediate bands in Fig. 6A, left panel), we can infer that the proposed NrnA binding patch (R262, R264, and R266) results in processive cleavage of nanoRNA substrates once they are bound. The R-x-R-x-R motif binding patch is short, and thus longer RNA substrates prefer binding NrnA in the opposite orientation, and are degraded from the 5′ end; this binding is likely weaker, and therefore, the exonuclease activity from the 5′ end is distributive, as evidenced by the intermediate bands present in Fig. 6B. As expected, mutating R262 and R264 to alanines abrogates the NrnA 3′ → 5′ activity, but does not affect 5′ → 3′ activity (Fig. 6B,C and Supplementary Fig. S4B). It is conceivable that absence of R262 and R264 in certain DHH family nanoRNases (*e.g*. Rv2873c and MSMEG_2630) dictates specificity for shorter, dinucleotide substrates.

Most nucleases have a fixed polarity, though a few dual polarity DNA exonucleases are known. Chase and Richardson reported that exonuclease VII is able to degrade DNA from both ends^32^. The RecBCD exonuclease/helicase can also degrade single-stranded DNA in both directions^33^. DHH family exonucleases are an interesting case where the polarity of an active site is modified by differential placement of substrate binding sites. While the overall architecture of DHH family enzymes remains mostly similar, nucleases with very different specificities have evolved by subtle variations in their nucleic acid binding patches.

Beyond nucleases, additional examples of nucleic acid enzymes with reversed polarities have been reported. Aquarius, a component of the human pre-mRNA intron-binding complex, is an SF1 superfamily RNA helicase with RNA unwinding activity opposite that of its central Upf1-like core^34^. The acquisition of several unique accessory domains fused to a conserved catalytic module may confer Aquarius with a reversed 3′ → 5′ polarity relative to the 5′ → 3′ Upf1^34^. Thg1, which adds a guanine to the 5′-end of some tRNAs, is another example of an enzyme that works in a direction (3′ → 5′) opposite that of all conventional polymerases^35^, even though Thg1 has an architecture similar to 5′ → 3′ DNA polymerases^36–38^.

Most bacterial exoribonucleases work 3′ → 5′, with *B. subtilis* RNase J1/J2^39, 40^ being the only other bacterial 5′ → 3′ RNase described to date. Here, we have shown that *Bacillus* NrnA has both 3′ → 5′ and 5′ → 3′ activities. To our knowledge, NrnA is a unique example of a bi-directional exonuclease that acts on RNA substrates. It is interesting to note that RecJ’s nuclease activity is stimulated by its additional OB domain and the protein SSB^31^, raising the prospect that NrnA’s 5′ → 3′ activity may be activated by an as yet undiscovered protein-protein interaction that alters its dimeric arrangement.

## Methods

Protein purification, crystallization, and data collection were performed as previously described^27^.

### Multiple sequence alignments

The sequence alignments were generated using T-Coffee^41^, and the secondary structures were added using ESpript^42^.

### Structure determination and refinement

Phases for the native apo-NrnA were obtained via molecular replacement with the program *PHASER* from the CCP4 suite^43^, using the polyalanine backbone of PDB ID: 3DEV (an uncharacterized protein from *S. haemolyticus* with 48% identity to NrnA) as a search model. A clear solution was seen in the *P*2_1_ space group with four monomers per asymmetric unit. After rigid body refinement, the structure was manually and reiteratively fitted in the electron density map using COOT^44^. Three rounds of automated protein building were run using the ARP/wARP software^45^, followed by several rounds of iterative model building. The structural models were refined with Refmac5^46^, and the final apo model has an *R* factor of 18.7% and an *R*-free of 23.7%.

Phases for the H103A-pAp complex, as well as all other co-crystal structures, were obtained via molecular replacement using the program *PHASER* from the CCP4 suite^43^ using a single N-terminal domain with a polyalanine backbone from the apo-NrnA structure as a search model. Initial models from the molecular replacement solutions were either constructed in ARP/wARP or by refinement against the complete apo-NrnA structure in Refmac5. The H103A-pAp structure was refined to a final *R* factor of 18.3% and an *R*-free of 23.5%. Electron density corresponding to the pAp ligand was built in COOT using a *Fo*-*Fc* difference map contoured at 3.0 *σ* and a 2*Fo*-*Fc* map contoured at 1.0 *σ*, followed by refinement in Refmac5. Data collection and refinement statistics are summarized for all structures in Supplementary Table S1.

A Polder omit map for the PDB ID: 5IPP structure was calculated in Phenix as described^29^ for the region surrounding F162. The Phenix default solvent exclusion radius of 5.0 Å was used. The map was contoured at 2.5 *σ* to observe planar density modeled as adenosine.

### Site-directed mutagenesis

The NrnA mutants were created by a one-step, QuikChange site-directed mutagenesis procedure (Stratagene Inc.). The resulting plasmids were verified by DNA sequencing. The NrnA mutants were overexpressed and purified as described for the WT enzyme^27^.

### 5′ labeled electrophoretic activity assays

For electrophoretic assays, the indicated oligoribonucleotide was 5′ labeled using [γ-^32^P]-ATP and T4 polynucleotide kinase. Reactions were carried out at 37 °C in either 30 or 50 μl volumes containing 50 mM Tris-HCl (pH 8.0), 150 mM NaCl, 2 mM MnCl_2_, 1 mM DTT (1X NrnA buffer), and the indicated concentration of substrate. Samples (4 μl) were removed at each time point and the reaction was terminated by addition of 2 volumes of gel loading buffer (95% formamide, 20 mM EDTA, 0.025% bromophenol blue, 0.025% xylene cyanol). Products were resolved on denaturing 7.5 M urea, 22.5% polyacrylamide gels (Sequa Gel; National Diagnostics Inc.) in the presence of 0.5X TBE buffer, and visualized using a STORM 840 PhosphorImager (Molecular Dynamics Inc.).

Dy647-A3 was chemically synthesized and PAGE purified (Dharmacon Inc). The molecule was deprotected and used in electrophoretic assays as described with the following modifications: NrnA concentration was increased to 2.85 μM and reactions were terminated with a modified gel loading buffer that lacked xylene cyanol (95% formamide, 20 mM EDTA, 0.025% bromophenol blue), as this molecule fluoresces at the DY647 excitation wavelength and also migrates the same distance as the intermediate degradation products. The 22.5% denaturing PAGE gels were placed on a Typhoon 9410 Trio (GE Healthcare Inc), and excited at 633 nm to image the DY647 moiety.

### 3′ labeled electrophoretic activity assays

The indicated A12 substrate was radiolabeled at its 3′ end with ^32^pCp (3000 Ci/mmol; PerkinElmer Inc.) by incubation with T4 RNA ligase (Ambion Inc.) for at least 1 hour at 37 °C or overnight at 25 °C, followed by heat inactivation at 65 °C for 20 minutes. The A12 substrate was further purified after labeling by ethanol precipitation (3 volumes of ethanol) in the presence of 1/10 volume of 3 M Na acetate pH 5.5. Exonuclease reactions were then carried out in either 30 μl or 50 μl volumes in 1X NrnA buffer using the indicated amount of enzyme and 3′ radiolabeled substrate. Samples (4 μl) were removed for each time point and terminated with 2 volumes of gel-loading buffer. Reaction products were resolved on denaturing polyacrylamide gels as described above.

## Electronic supplementary material

Supplementary Information

## References

[1] Goldman SR, Ebright RH, Nickels BE (2009). Direct detection of abortive RNA transcripts *in vivo*. Science.

[2] Goldman SR (2011). NanoRNAs prime transcription initiation *in vivo*. Mol. Cell.

[3] Nickels BE, Dove SL (2011). NanoRNAs: a class of small RNAs that can prime transcription initiation in bacteria. J. Mol. Biol..

[4] Rao F (2010). YybT is a signaling protein that contains a cyclic dinucleotide phosphodiesterase domain and a GGDEF domain with ATPase activity. J. Biol. Chem..

[5] Cohen D (2015). Oligoribonuclease is a central feature of cyclic diguanylate signaling in *Pseudomonas aeruginosa*. Proc. Natl. Acad. Sci. USA.

[6] Orr MW (2015). Oligoribonuclease is the primary degradative enzyme for pGpG in *Pseudomonas aeruginosa* that is required for cyclic-di-GMP turnover. Proc. Natl. Acad. Sci. USA.

[7] He Q (2016). Structural and biochemical insight into the mechanism of Rv2837c from *Mycobacterium tuberculosis* as a c-di-NMP phosphodiesterase. J. Biol. Chem..

[8] Nickels BE (2012). A new way to start: nanoRNA-mediated priming of transcription initiation. Transcription.

[9] Vvedenskaya IO (2012). Growth phase-dependent control of transcription start site selection and gene expression by nanoRNAs. Genes Dev..

[10] Druzhinin SY (2015). A conserved pattern of primer-dependent transcription initiation in *Escherichia coli* and *Vibrio cholerae* revealed by 5′ RNA-seq. PLoS Genet..

[11] Ghosh S, Deutscher MP (1999). Oligoribonuclease is an essential component of the mRNA decay pathway. Proc. Natl. Acad. Sci. USA.

[12] Niyogi SK, Datta AK (1975). A novel oligoribonuclease of *Escherichia coli*. I. Isolation and properties. J. Biol. Chem..

[13] Datta AK, Niyogi K (1975). A novel oligoribonuclease of *Escherichia coli*. II. Mechanism of action. J. Biol. Chem..

[14] Zhang X, Zhu L, Deutscher MP (1998). Oligoribonuclease is encoded by a highly conserved gene in the 3′-5′ exonuclease superfamily. J. Bacteriol..

[15] Zuo Y, Deutscher MP (2001). Exoribonuclease superfamilies: structural analysis and phylogenetic distribution. Nucleic Acids Res..

[16] Mechold U, Fang G, Ngo S, Ogryzko V, Danchin A (2007). YtqI from *Bacillus subtilis* has both oligoribonuclease and pAp-phosphatase activity. Nucleic Acids Res..

[17] Fang M (2009). Degradation of nanoRNA is performed by multiple redundant RNases in *Bacillus subtilis*. Nucleic Acids Res..

[18] Postic G, Danchin A, Mechold U (2012). Characterization of NrnA homologs from *Mycobacterium tuberculosis* and *Mycoplasma pneumoniae*. RNA.

[19] Aravind. L, Koonin EV (1998). A novel family of predicted phosphoesterases includes *Drosophila* prune protein and bacterial RecJ exonuclease. Trends Biochem. Sci..

[20] Guo M (2009). The C-Ala domain brings together editing and aminoacylation functions on one tRNA. Science.

[21] Yamagata A, Kakuta Y, Masui R, Fukuyama K (2002). The crystal structure of exonuclease RecJ bound to Mn^2+^ ion suggests how its characteristic motifs are involved in exonuclease activity. Proc. Natl. Acad. Sci. USA.

[22] Wakamatsu T (2010). Structure of RecJ exonuclease defines its specificity for single-stranded DNA. J. Biol. Chem..

[23] Wakamatsu T (2011). Role of RecJ-like protein with 5′-3′ exonuclease activity in oligo(deoxy)nucleotide degradation. J. Biol. Chem..

[24] Uemura Y (2013). Crystal structure of the ligand-binding form of nanoRNase from *Bacteroides fragilis*, a member of the DHH/DHHA1 phosphoesterase family of proteins. FEBS Letters.

[25] Srivastav R (2014). Unique Subunit packing in *mycobacterial* nanoRNase leads to alternate substrate recognitions in DHH phosphodiesterases. Nucleic Acids Res..

[26] Cheng K (2016). Structural basis for DNA 5′-end resection by RecJ. Elife.

[27] Nelersa CM, Schmier BJ, Malhotra A (2011). Purification and crystallization of *Bacillus subtilis* NrnA, a novel enzyme involved in nanoRNA degradation. Acta Crystallogr. Sect. F Struct. Biol. Cryst. Commun..

[28] Ahn S (2001). The “open” and “closed” structures of the type-C inorganic pyrophosphatases from *Bacillus subtilis* and *Streptococcus gordonii*. J. Mol. Biol..

[29] Liebschner D (2017). Polder maps: improving OMIT maps by excluding bulk solvent. Acta Crystallogr. D Struct. Biol..

[30] Lovett ST, Kolodner RD (1989). Identification and purification of a single-stranded-DNA-specific exonuclease encoded by the recJ gene of *Escherichia coli*. Proc. Natl. Acad. Sci. USA.

[31] Han ES (2006). RecJ exonuclease: substrates, products and interaction with SSB. Nucleic Acids Res..

[32] Chase JW, Richardson CC (1974). Exonuclease VII of *Escherichia coli*. Mechanism of action. J. Biol. Chem..

[33] Wang J, Chen R, Julin DA (2000). A single nuclease active site of the *Escherichia coli* RecBCD enzyme catalyzes single-stranded DNA degradation in both directions. J. Biol. Chem..

[34] De I (2015). The RNA helicase Aquarius exhibits structural adaptations mediating its recruitment to spliceosomes. Nat. Struct. Mol. Biol..

[35] Jackman JE, Gott JM, Gray MW (2012). Doing It in Reverse: 3′-to-5′ Polymerization by the Thg1 Superfamily. RNA..

[36] Hyde SJ (2010). tRNA(His) guanylyltransferase (THG1), a unique 3′-5′ nucleotidyl transferase, shares unexpected structural homology with canonical 5′-3′ DNA polymerases. Proc. Natl. Acad. Sci. USA.

[37] Nakamura A (2013). Structural basis of reverse nucleotide polymerization. Proc. Natl. Acad. Sci. USA.

[38] Perona JJ, Oza JP (2010). Crystal structure of a reverse polymerase. Proc. Natl. Acad. Sci. USA.

[39] Mathy N (2010). *Bacillus subtilis* ribonucleases J1 and J2 form a complex with altered enzyme behaviour. Mol. Microbiol..

[40] Dorléans A (2011). Molecular basis for the recognition and cleavage of RNA by the bifunctional 5′–3′ exo/endoribonuclease RNase. J. Structure.

[41] Notredame C, Higgins DG, Heringa J (2000). T-Coffee: A novel method for fast and accurate multiple sequence alignment. J. Mol. Biol..

[42] Gouet P, Courcelle E, Stuart DI, Métoz F (1999). ESPript: analysis of multiple sequence alignments in PostScript. Bioinformatics.

[43] Winn MD (2011). Overview of the CCP4 suite and current developments. Acta Crystallogr. D Biol. Crystallogr..

[44] Emsley P, Cowtan K (2004). Coot: model-building tools for molecular graphics. Acta Crystallogr. D Biol. Crystallogr..

[45] Langer G, Cohen SX, Lamzin VS, Perrakis A (2008). Automated macromolecular model building for X-ray crystallography using *ARP/wARP* version 7. Nature Protocols.

[46] Murshudov GN (2011). REFMAC5 for the refinement of macromolecular crystal structures. Acta Crystallogr. D Biol. Crystallogr..

[47] DeLano, W. L. The PyMOL Molecular Graphics System (2002).

[48] Stierand K, Maass PC, Rarey M (2006). Molecular complexes at a glance: automated generation of two-dimensional complex diagrams. Bioinformatics.

