# Supplementary material for: Structural Basis for the Bidirectional Activity of Bacillus nanoRNase NrnA
Source: Sci Rep. 2017 Sep 11;7:11085. doi: 10.1038/s41598-017-09403-x (PMC5593865; doi:10.1038/s41598-017-09403-x)
Supplement: Supplementary file 1 — Supplementary Information [file 41598_2017_9403_MOESM1_ESM.pdf]

Supplementary Information

Structural Basis for the Bidirectional Activity of *Bacillus* nanoRNase NrnA

Brad J. Schmier[1,2,*], Claudiu M. Nelersa[1,*], and Arun Malhotra[1]

[1] Department of Biochemistry and Molecular Biology, University of Miami Miller School of Medicine, PO Box 016129, Miami, FL, 33101-6129, USA

[2] Present Address: Molecular Biology Program, Sloan-Kettering Institute, New York, NY 10065, USA.

[*] These authors contributed equally to this work.

Corresponding Author: Arun Malhotra: Ph: (305) 243-2826; Fax: (305) 243-3955; Email: amalhotra@miami.edu

Figure S1

(A)

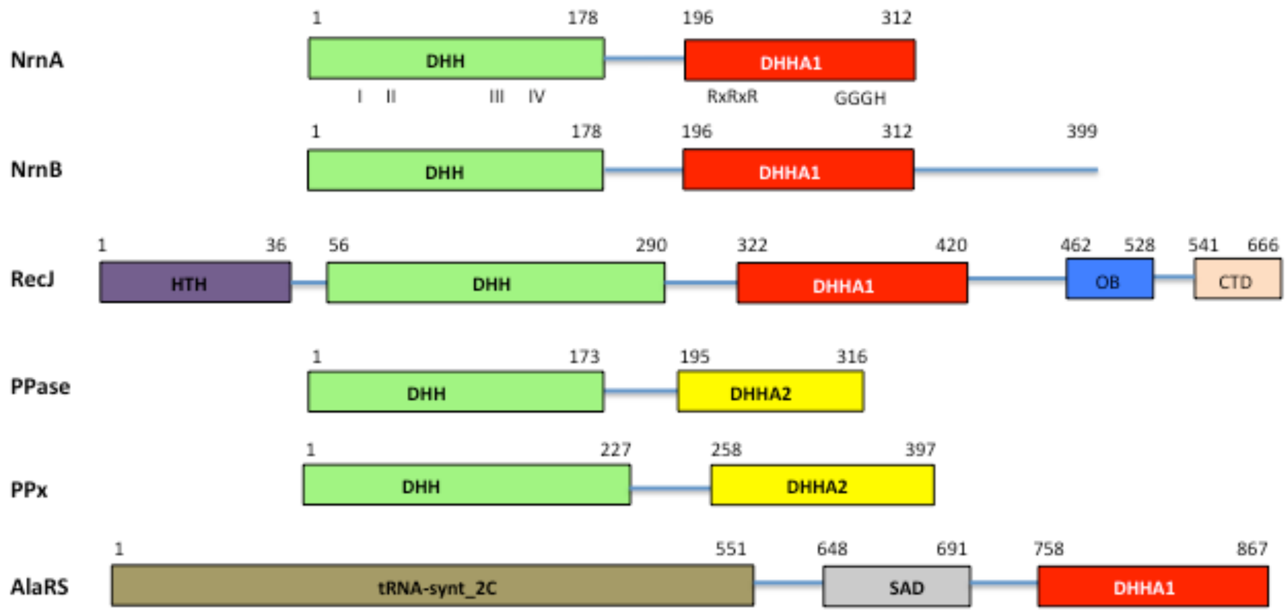

Fig. S1. Domain Structure and Sequence Analysis of NrnA. (A) Linear domain map of selected DHH family proteins. The domains are colored as follows: DHH, light green; DHHA1, red; DHHA2; yellow; other accessory domains, varied colors. The listed DHH orthologs are NrnA and NrnB from *B. subtilis;* RecJ from *T. thermophilus* (PDB ID: 2ZXP)*; S. cerevisiae* exopolyphosphatase (PPx; PDB ID: 2QB7); *B. subtilis* inorganic pyrophosphatase (PPase; PDB ID: 1K23); *A. aeolicus* alanyl-tRNA synthetase (AlaRS; PDB ID: 3G98).

(B) Multiple sequence alignment of selected NrnA orthologs. Sequences are from the UniProt non-redundant protein sequence database. *Bacillus subtilis* NrnA NRNA_BACSU (UniProt accession no. O34600) is aligned with several putative bacterial nanoRNases, including NrnB. The orthologs listed are: Q8Y6V6 *Listeria monocytogenes* (Q8Y6V6_LISMO); B2DY69 *Streptococcus pneumoniae* (B2DY69_STRPN); Q5FLW8 *Lactobacillus acidophilus* (Q5FLW8_LACAC); Q4L745 *Staphylococcus haemolyticus* strain JCSC1435 (Q4L745_STAHJ); P22746.3 *Mycoplasma genitalium* (MGP1_MYCGE); and O31824 *Bacil*lus *subtilis* NrnB (O31824_BACSU). NrnB has an extended C-terminal tail that is not seen in other NrnA orthologs, and its final 45 residues are not shown. Sequence alignments were generated using T-Coffee (41), and secondary structure elements from the *Bacillus* NrnA structure (Fig. 1C) were added using ESPript (42).

Figure S1

(B)

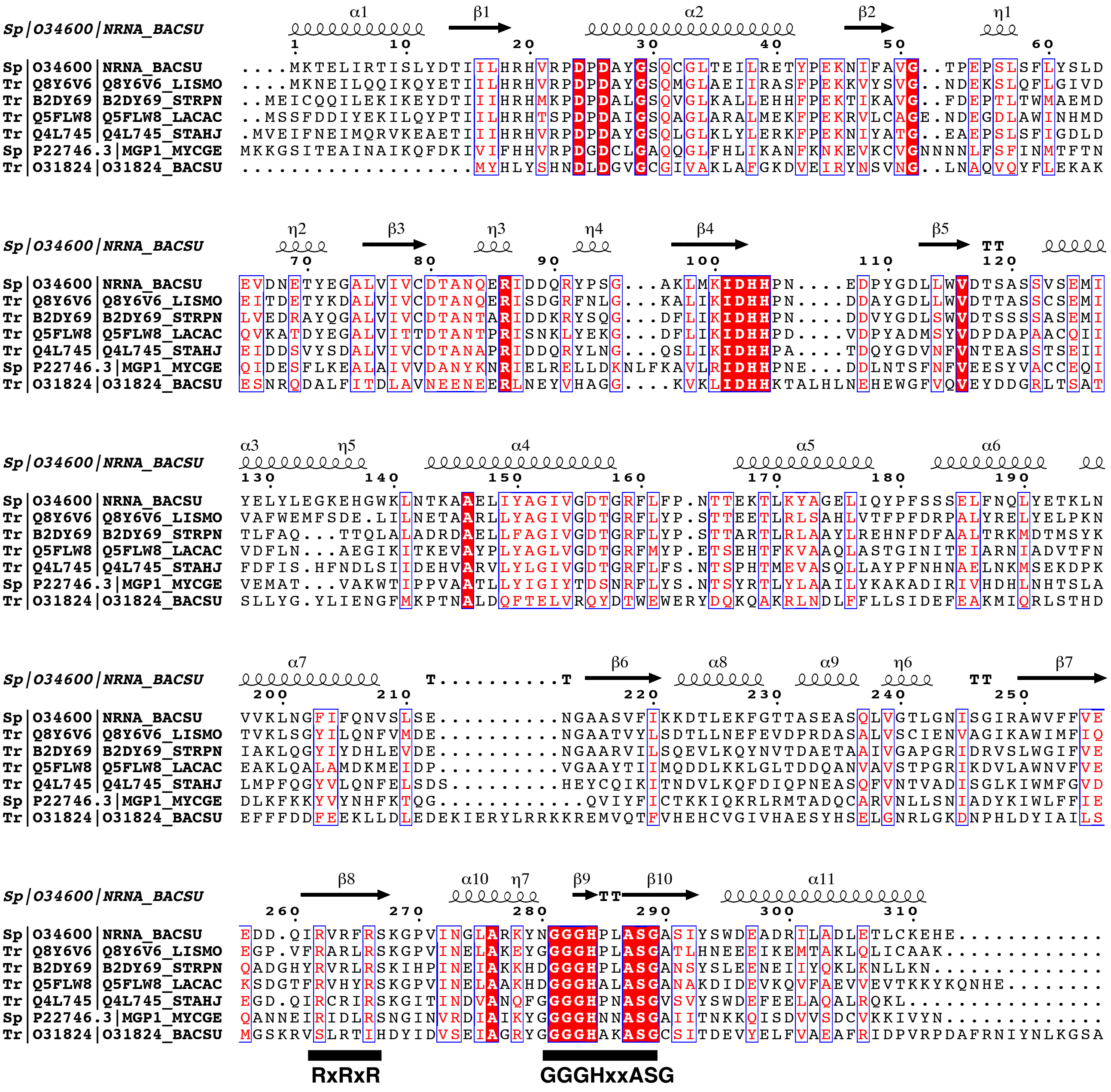
Sp|O34600|NRNA_BACSU
α1 β1 α2 β2 η1
1 10 20 30 40 50 60
Sp|O34600|NRNA_BACSU ....MKTELIRTISLYDTIILHRHVRPDPDAYGSQCGLTEILRETYPEKNIFAVG..TPEPSLSFLYSLD
Tr|Q8Y6V6|Q8Y6V6_LISMO ....MKNEILQQIKQYETIILHRHVRPDPDAYGSQMGLAEIIRASFPEKKVYSVG..NDEKSLQFLGIVD
Tr|B2DY69|B2DY69_STRPN ..MEICQQILEKIKEYDTIIIHRHMKPDPDALGSQVGLKALLEHHFPEKTIKAVG..FDEPTLTWMAEMD
Tr|Q5FLW8|Q5FLW8_LACAC ..MSSFDDIYEKILQYPTIILHRHTSPDPDAIGSQAGLARALMEKFPEKRVLCAGE.NDEGDLAWINHMD
Tr|Q4L745|Q4L745_STAHJ .MVEIFNEIMQRVKEAETIIIHRHVRPDPDAYGSQLGLKLYLERKFPEKNIYATG..EAEPSLSFIGDLD
Sp|P22746.3|MGP1_MYCGE MKKGSITEAINAIKQFDKIVIFHHVRPDGDCLGAQQGLFHLIKANFKNKEVKCVGNNNNLFSFINMTFTN
Tr|O31824|O31824_BACSU ...................MYHLYSHNDLDGVGCGIVAKLAFGKDVEIRYNSVNG..LNAQVQYFLEKAK
Sp|O34600|NRNA_BACSU
η2 β3 η3 η4 β4 β5 TT
70 80 90 100 110 120
Sp|O34600|NRNA_BACSU EVDNETYEGALVIVCDTANQERIDDQRYPSG...AKLMKIDHHPN....EDPYGDLLWVDTSASSVSEMI
Tr|Q8Y6V6|Q8Y6V6_LISMO EITDETYKDALVIVCDTANQERISDGRFNLG...KALIKIDHHPN....DDAYGDLLWVDTTASSCSEMI
Tr|B2DY69|B2DY69_STRPN LVEDRAYQGALVIVCDTANTARIDDKRYSQG...DFLIKIDHHPN....DDVYGDLSWVDTSSSSASEMI
Tr|Q5FLW8|Q5FLW8_LACAC QVKATDYEGALVITTDTANTPRISNKLYEKG...DFLIKIDHHPD....VDPYADMSYVDPDAPAACQII
Tr|Q4L745|Q4L745_STAHJ EIDDSVYSDALVIVCDTANAPRIDDQRYLNG...QSLIKIDHHPA....TDQYGDVNFVNTEASSTSEII
Sp|P22746.3|MGP1_MYCGE QIDESFLKEALAIVVDANYKNRIELRELLDKNLFKAVLRIDHHPNE...DDLNTSFNFVEESYVACCEQI
Tr|O31824|O31824_BACSU ESNRQDALFITDLAVNEENEERLNEYVHAGG....KVKLIDHHKTALHLNEHEWGFVQVEYDDGRLTSAT
Sp|O34600|NRNA_BACSU
α3 η5 α4 α5 α6
130 140 150 160 170 180 190
Sp|O34600|NRNA_BACSU YELYLEGKEHGWKLNTKAAELIYAGIVGDTGRFLFP.NTTEKTLKYAGELIQYPFSSSELFNQLYETKLN
Tr|Q8Y6V6|Q8Y6V6_LISMO VAFWEMFSDE.LILNETAARLLYAGIVGDTGRFLYP.STTEETLRLSAHLVTFPFDRPALYRELYELPKN
Tr|B2DY69|B2DY69_STRPN TLFAQ...TTQLALADRDAELLFAGIVGDTGRFLYP.STTARTLRLAAYLREHNFDFAALTRKMDTMSYK
Tr|Q5FLW8|Q5FLW8_LACAC VDFLN...AEGIKITKEVAYPLYAGLVGDTGRFMYP.ETSEHTFKVAAQLASTGINITEIARNIADVTFN
Tr|Q4L745|Q4L745_STAHJ FDFIS.HFNDLSIIDEHVARVLYLGIVGDTGRFLFS.NTSPHTMEVASQLLAYPFNHNAELNKMSEKDPK
Sp|P22746.3|MGP1_MYCGE VEMAT...VAKWTIPPVAATLLYIGIYTDSNRFLYS.NTSYRTLYLAAILYKAKADIRIVHDHLNHTSLA
Tr|O31824|O31824_BACSU SLLYG.YLIENGFMKPTNALDQFTELVRQYDTWEWERYDQKQAKRLNDLFFLLSIDEFEAKMIQRLSTHD
Sp|O34600|NRNA_BACSU
α7 T..........T β6 α8 α9 η6 TT β7
200 210 220 230 240 250
Sp|O34600|NRNA_BACSU VVKLNGFIFQNVSLSE..........NGAASVFIKKDTLEKFGTTASEASQLVGTLGNISGIRAWVFFVE
Tr|Q8Y6V6|Q8Y6V6_LISMO TVKLSGYILQNFVMDE..........NGAATVYLSDTLLNEFEVDPRDASALVSCIENVAGIKAWIMFIQ
Tr|B2DY69|B2DY69_STRPN IAKLQGYIYDHLEVDE..........NGAARVILSQEVLKQYNVTDAETAAIVGAPGRIDRVSLWGIFVE
Tr|Q5FLW8|Q5FLW8_LACAC EAKLQALAMDKMEIDP..........VGAAYTIIMQDDLKKLGLTDDQANVAVSTPGRIKDVLAWNVFVE
Tr|Q4L745|Q4L745_STAHJ LMPFQGYVLQNFELSDS.........HEYCQIKITNDVLKQFDIQPNEASQFVNTVADISGLKIWMFGVD
Sp|P22746.3|MGP1_MYCGE DLKFKKYVYNHFKTQG............QVIYFICTKKIQKRLRMTADQCARVNLLSNIADYKIWLFFIE
Tr|O31824|O31824_BACSU EFFFDDFEEKLLDLEDEKIERYLRRKKREMVQTFVHEHCVGIVHAESYHSELGNRLGKDNPHLDYIAILS
Sp|O34600|NRNA_BACSU
β8 α10 η7 β9 TT β10 α11
260 270 280 290 300 310
Sp|O34600|NRNA_BACSU EDD.QIRVRFRSKGPVINGLARKYNGGGHPLASGASIYSWDEADRILADLETLCKEHE...........
Tr|Q8Y6V6|Q8Y6V6_LISMO EGP.VFRARLRSKGPVINELAKEYGGGGHPLASGATLHNEEEIKEMTAKLQLICAAK.............
Tr|B2DY69|B2DY69_STRPN QADGHYRVRLRSKIHPINEIAKKHDGGGHPLASGANSYSLEENEIIYQKLKNLLKN..............
Tr|Q5FLW8|Q5FLW8_LACAC KSDGTFRVHYRSKGPVINELAAKHDGGGHALASGANAKDIDEVKQVFAEVVEVTKKYKQNHE........
Tr|Q4L745|Q4L745_STAHJ EGD.QIRCRIRSKGITINDVANQFGGGGHPNASGVSVYSWDEFEELAQALRQKL................
Sp|P22746.3|MGP1_MYCGE QANNEIRIDLRSNGINVRDIAIKYGGGGHNNASGAIITNKKQISDVVSDCVKKIVYN.............
Tr|O31824|O31824_BACSU MGSKRVSLRTIHDYIDVSEIAGRYGGGGHAKASGCSITDEVYELFVAEAFRIDPVRPDAFRNIYNLKGSA
RxRxR
GGGHxxASG

Figure S2

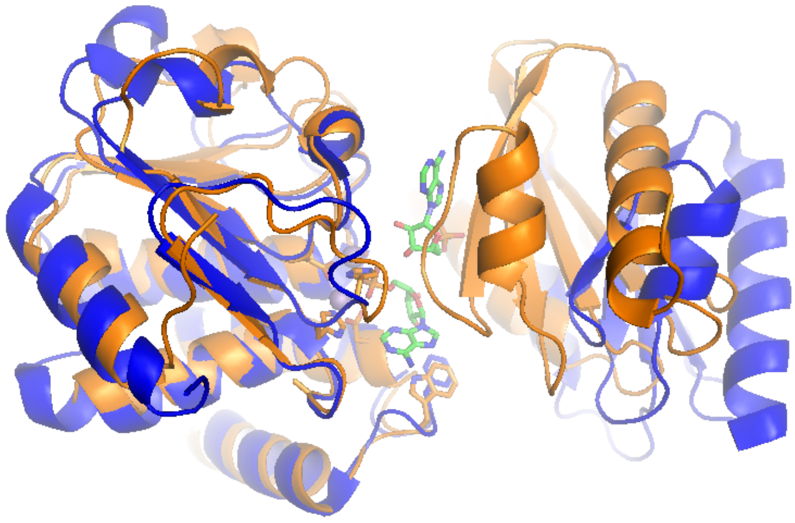

Fig. S2. Motion of the DHHA1 Domain. Structural alignment of the DHH domains from *B. subtilis* NrnA (blue) and *M. tuberculosis* Rv2837c (orange). Rv2837C is bound to two AMP molecules after cleavage of a linear dinucleotide (7). The DHHA1 domain of Rv2837c appears in the immediate post-cleavage, closed conformation of the enzyme. Alignments were carried out in Pymol (47).

Figure S3

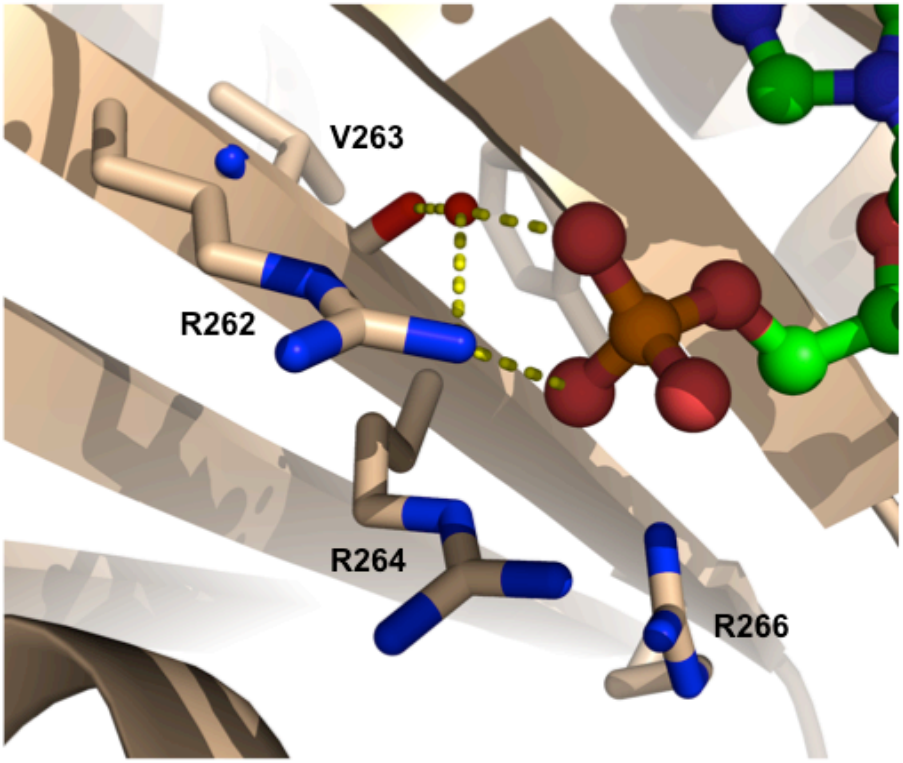

Fig. S3. NrnA interactions with substrate. View of a bound water molecule mediating a H-bond network involving V263, R262, and the 5'-phosphate of pAp bound on the DHHA1 domain of NrnA.

Figure S4

(A)

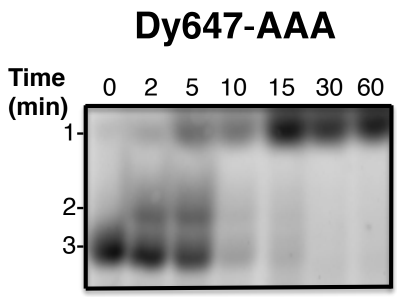

Fig. S4 (A). NrnA Shows Distributive 3'→ 5' Activity on 5' Blocked NanoRNA Substrate. WT NrnA (2.85 µM) was assayed in a time course experiment with a trinucleotide RNA substrate blocked at the 5' end with the fluorescent dye DY647 (DY647-A3, 1 µM, Dharmacon). Reactions products were stopped with a modified gel loading buffer (95% formamide, 20 mM EDTA, 0.025% bromophenol blue) that lacked xylene cyanol, as this molecule fluoresces at the DY647 excitation wavelength, and loaded on a 22.5% denaturing PAGE. Due to the large, neutral mass of DY647 relative to the nanoRNA, intermediate degradation products run up the gel relative to the starting material. The gel was visualized on a Typhoon 9410 Trio, and excited at 633 nm to image the DY647 fluorescent moiety.

(B)

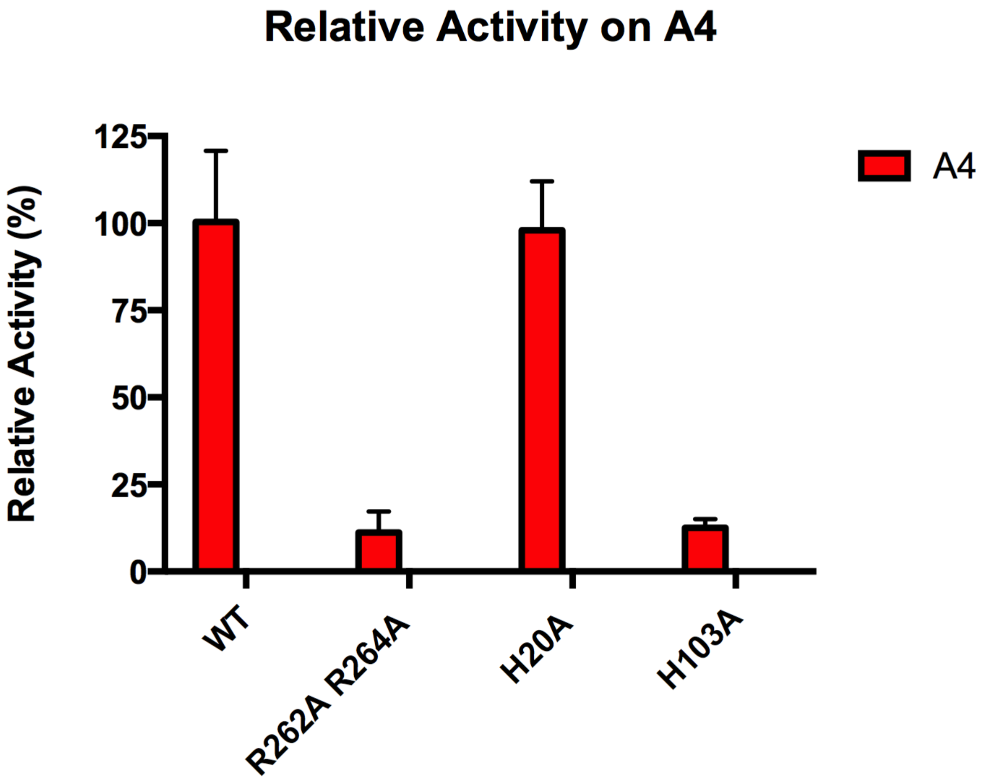

Fig. S4 (B). Relative activity of NrnA mutants on an A4 nanoRNA substrate. Figure shows % appearance of the product (labeled mononucleotide) after a fixed time point in the linear range of the WT enzyme (normalized as 100%). Average of three independent experiments is shown.

Figure S5

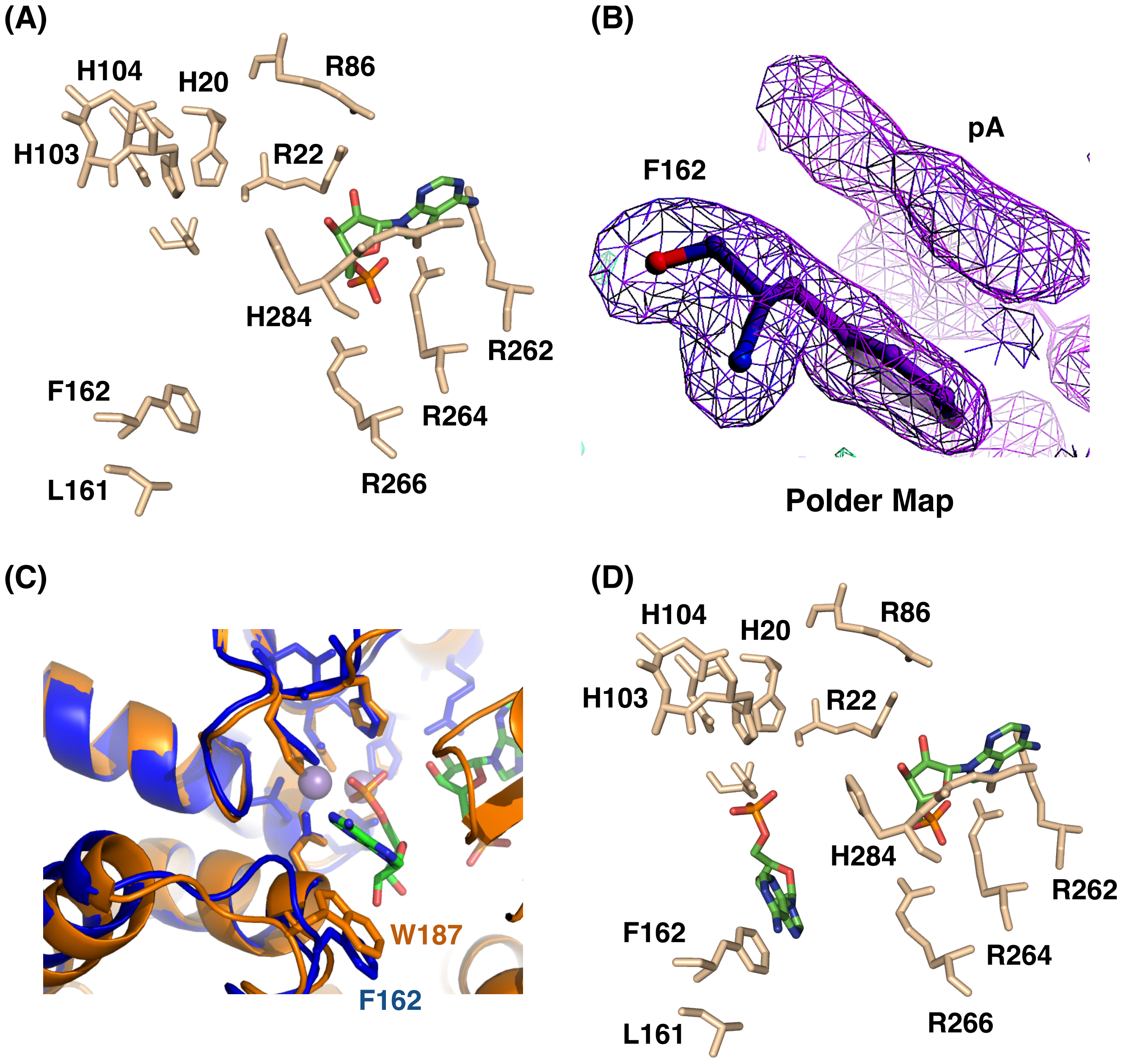

Fig. S5. Structure of NrnA bound to mononucleotides. (A) Structure of NrnA H103A:pAA*A (PDB ID: 5IPP) is colored wheat and shows a stick representation of active site residues H103, H104, H20 along with binding site residues R262, R264, R266, and H284. R266 and R264 coordinate the 5'-phosphate of the bound pA mononucleotide. The F162 residue proposed to play a role in base stacking is also shown. **(B)** Polder omit map of region surrounding F162 with density contoured at 2.5 σ. The map was calculated in Phenix as described (29). The observed planar density is labeled as a pA mononucleotide. Image was created in Coot (44). **(C)** Structure based alignment of the active sites of *B. subtilis* NrnA (blue) and *M. tuberculosis* Rv2873c (orange). The role of W187 in base stacking and its structural equivalency to F162 is highlighted. The two pA mononucleotides bound to Rv2873c are colored by atom type. **(D)** Structure of NrnA H103A:pAA*A in the same orientation as (A) with the pA mononucleotide from Rv2873c modeled at the site of the Polder map density described in (B).

Figure S6

(A)

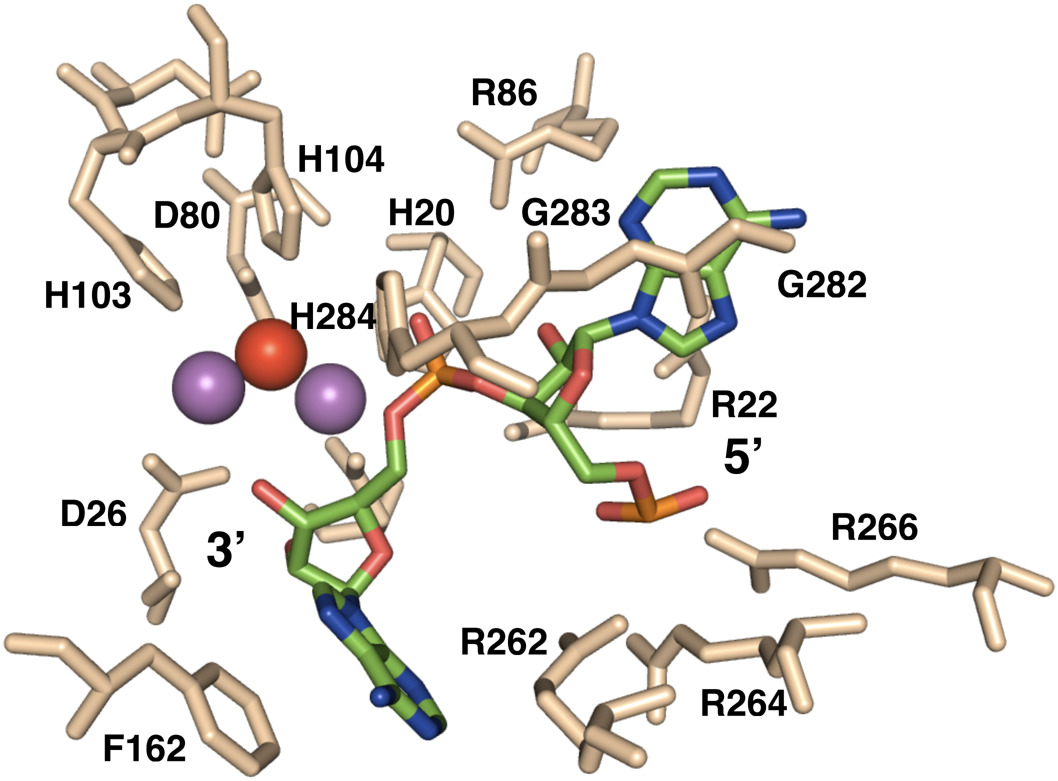

R86
H104
D80
H20
G283
G282
H103
H284
R22
5’
D26
3’
R266
R262
R264
F162

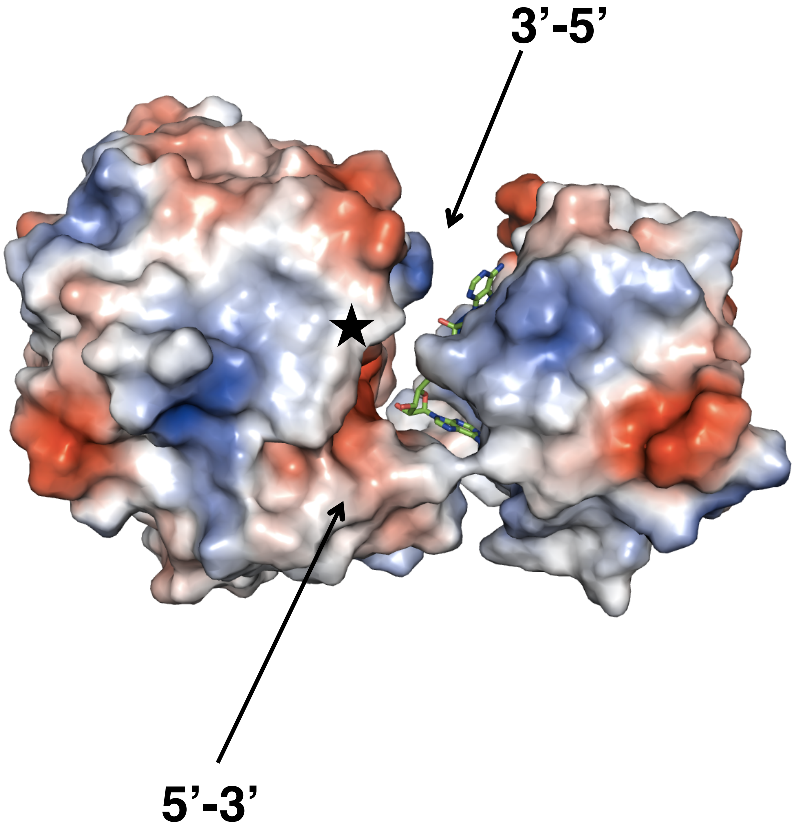

3’-5’
5’-3’

Figure S6

(B)

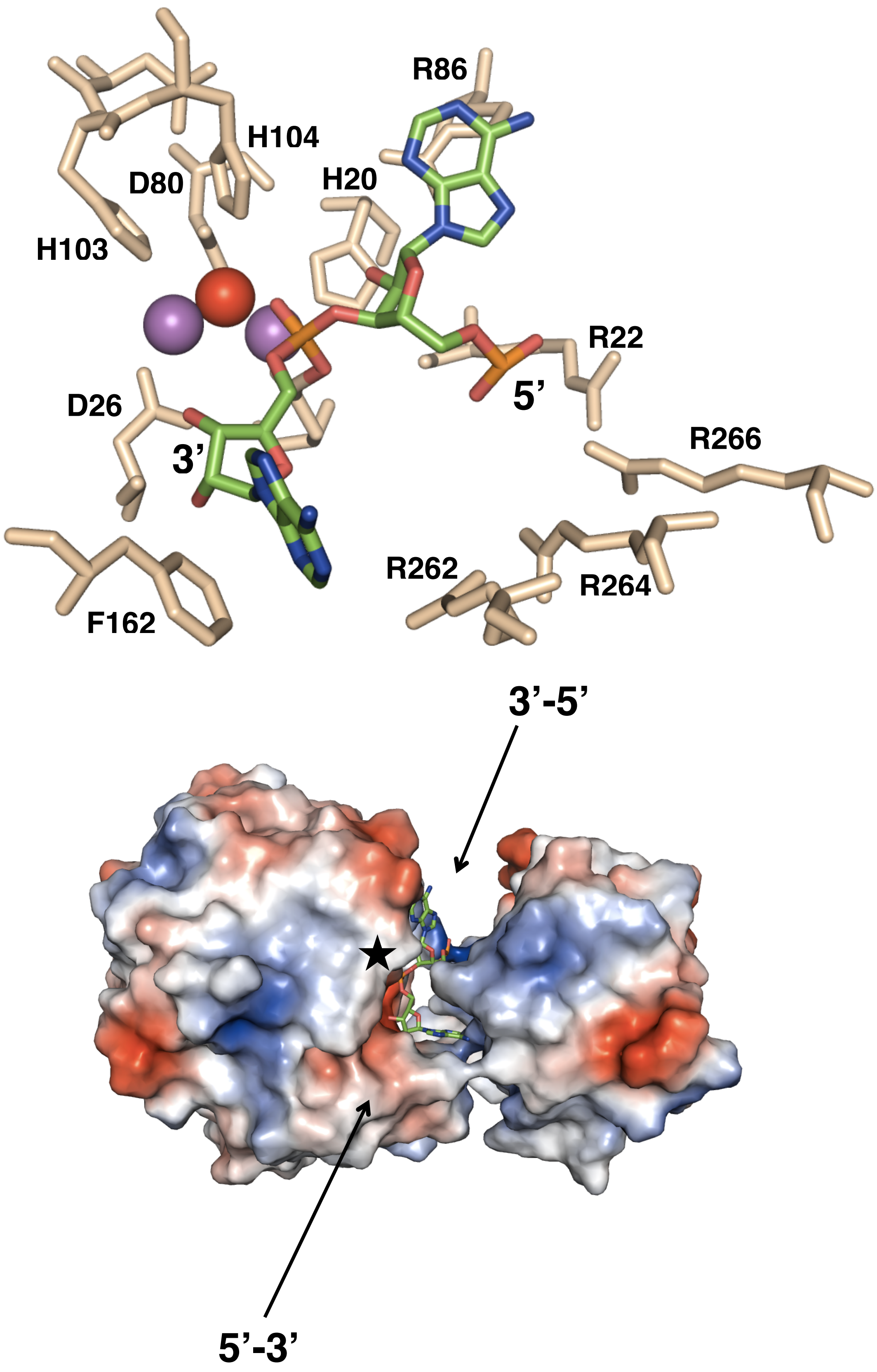

Figure S6

(C)

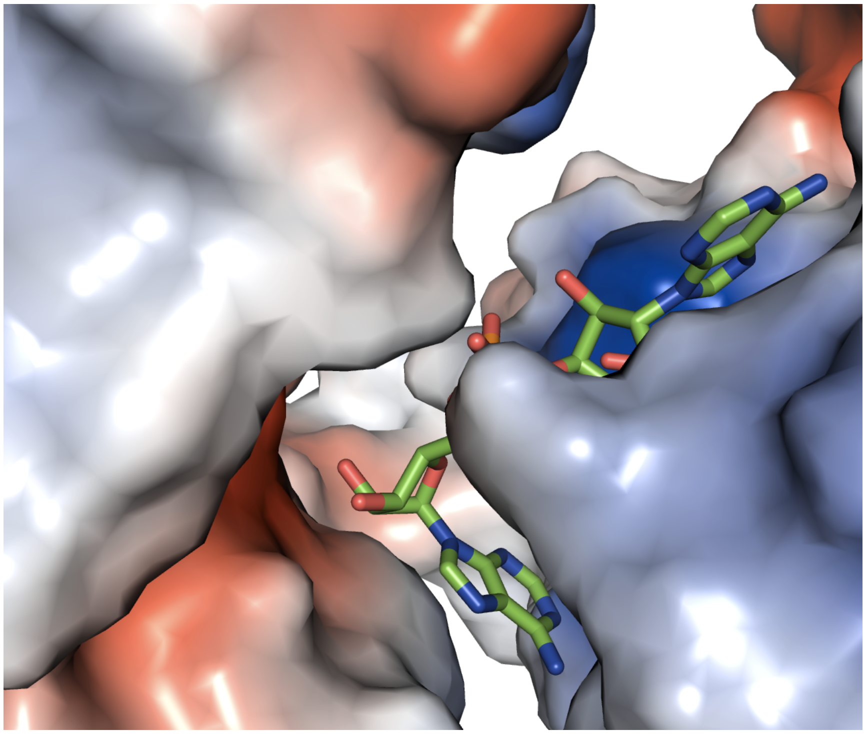

Fig. S6. NanoRNA substrate modeled into NrnA during the binding and catalytic steps. (A) NrnA structure (H103A:pAp) with the dinucleotide pApA modeled at the nanoRNA binding site. The 3' ribose and base were modeled at the end of the observed coordinates of pAp in the co-crystal structure. Top panel shows a stick representation with the protein colored wheat, and the bound dinucleotide colored by atom type. Selected NrnA residues from the active site, the GGGH motif, and the R-x-R-x-R motif are shown. The bottom panel shows the molecular accessible surface colored by electrostatic charge, with the modeled RNA substrate in a stick representation. The 3'→5' and 5'→3' substrate-binding paths are highlighted by arrows, and the approximate position of the active site is indicated by a star. While active metal ions are shown (purple spheres and a bound water in red) to identify the active site, this co-crystal structure did not include any metal and no metal ions were seen. This structure likely represents the binding step in nanoRNA hydrolysis. **(B)** NrnA structure (H103A:Mn:pAp) with the dinucleotide pApA modeled at the catalytic step. Metal ions seen in this structure are shown as purple spheres. The dinucleotide scissile phosphate is positioned at the site of the cleaved 3' phosphate observed in the co-crystal structure (see Fig. 4). The GGGH motif has been removed for clarity. **(C)** A zoomed-in view of the substrate bound on NrnA from (A). Figures were created in Pymol (47).

Figure S6

(D)

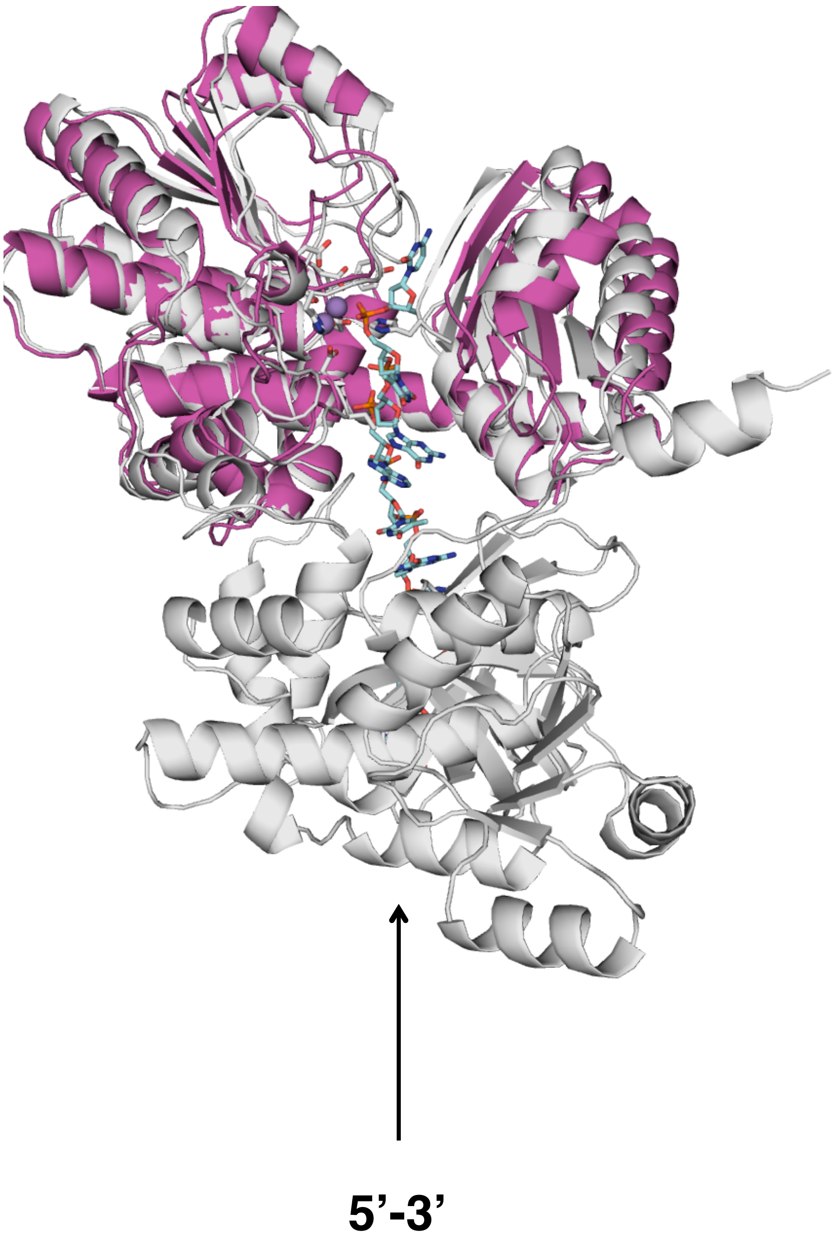

Fig. S6. (D) Structure of the *D. radiodurans* RecJ (grey) bound to single-strand DNA (PDB ID: 5F56). The 5’→3’ substrate-binding path is indicated and the active site is indicated by a star. The DHH and DHHA1 domains are aligned to the same domains of the *T. thermophilus* RecJ structure (magenta) (PDB ID: 1IR6). Figures were created in Pymol (47).

Table S1. Data collection and refinement statistics

PDB ID	5IUF	5IZO	5IPP	5J21
	**H103A:pAp**	**H103A:Mn:pAp**	**H103A:pAA*A**	**Native**
**Data Collection**				
Wavelength (Å)	0.97918	0.97918	0.97918	0.97918
Space group	$P2_1$	$P2_1$	$P2_1$	$P2_1$
Cell dimensions				
a, *b*, *c* (Å)	50.5, 121.3, 123.7	50.7, 119.6, 124.1	51.03, 125.2, 116.8	50.62, 121.3, 123.4
α, β, γ (°)	90.0, 91.7, 90.0	90.0, 91.6, 90.0	90.0, 90.2, 90.0	90.0, 91.3, 90.0
Resolution (Å)	46.62-1.95 (2.02-1.95)	50.00-1.95 (2.02-1.95)	46.82-1.95 (2.02-1.95)	46.44-2.00 (2.03-2.00)
R_{merge}	0.085 (0.470)	0.090 (0.436)	0.074 (0.446)	0.058 (0.315)
$I/\sigma I$	17.88 (2.00)	17.44 (2.18)	16.44 (1.87)	35.19 (4.5)
Completeness (%)	98.1 (95.2)	93.3 (80.0)	97.6 (94.3)	98.3 (96.9)
Redundancy	3.6	3.4	3.0	5.0
No. reflections				
Total	380,704	343,384	316,215	493,314
Unique	107,136	106,608	98,753	93,522
**Refinement**				
Resolution (Å)	1.95 (2.00-1.95)	1.95 (2.00-1.95)	1.95 (2.00-1.95)	2.00 (2.05-2.00)
R_{work}/R_{free} (%)	17.5/21.6(30.2/31.6)	21.2/26.7(31.4/33.8)	18.6/23.8 (26.2/30.5)	18.3/23.2 (25.3/30.4)
No. atoms				
Protein	9896	9534	9918	9748
Solvent (water/ion)	840	523	760	684
Ligand	54	20	46	-
Metals	-	8	-	-
B-factors (Å^2)				
Protein	33.57	41.97	23.01	31.57
Solvent	44.64	52.65	41.01	44.21
Ligand	51.58	56.07	56.75	-
Metals	-	39.80	-	-
R.M.S. deviations				
Bond lengths (Å)	0.019	0.022	0.024	0.027
Bond angles (°)	1.891	1.899	1.885	2.072

$R_{merge} = (\Sigma|I-\langle I\rangle|)/(\Sigma\langle I\rangle)$, where $\langle I\rangle$ refers to the average intensity of multiple measurements of the same reflection. *R* and R_{free} = $(\Sigma|F_{obs}-F_{cal}|)/(\Sigma|F_{obs}|)$. R_{free} was calculated using 5% of the data not included in the refinement.
